# Mitigating request flooding attack in named data networking using federated learning

**DOI:** 10.1038/s41598-026-58988-9

**Published:** 2026-06-24

**Authors:** Mohamed Lamine Benmaidi, Nasreddine Lagraa, Bouziane Brik, Lotfi Jlali

**Affiliations:** 1 LIM Laboratory, Amar Telidji University of Laghouat, Bp 37G, Ghardaia Road, Laghouat, 03000 Algeria; 2 Computer Science Department, College of Computing and Informatics, University of Sharjah, UAE; 3 Department of Mathematics and Statistics, College of Science, Imam Mohammad Ibn Saud Islamic University (IMSIU), Riyadh, 11432 Saudi Arabia

**Keywords:** Engineering, Mathematics and computing

## Abstract

Named Data Networking (NDN) represents a paradigm shift toward content-centric architectures but remains critically vulnerable to Interest Flooding Attacks (IFAs), where malicious actors overwhelm router Pending Interest Tables with spurious requests, causing service degradation and denial-of-service. To address the limitations of existing approaches, including high false positives in threshold-based methods and substantial overhead in centralized learning, we propose FL-IFAshield, a novel federated learning framework for adaptive IFA mitigation. Our solution integrates dynamic Poisson-EMA thresholding for accurate flood detection, entropy-aware federated aggregation to handle non-IID traffic distributions across edge routers, and Byzantine-robust mechanisms with differential privacy guarantees. Comprehensive evaluation on the FIT/IoT-LAB testbed with 100 routers demonstrates exceptional performance: 93.1% F1-score in attack detection, only 5% false positives, 28 ms average end-to-end latency ($\Delta t_{\text {e2e}}$), and over 90% legitimate Interest Satisfaction Ratio under sophisticated collusive attacks, while maintaining minimal computational overhead (<9% CPU utilization on ARMv8 routers). FL-IFAshield significantly improves security performance, offering 35% higher accuracy than static thresholding and 60% lower communication overhead than centralized approaches. While simpler heuristic baselines naturally incur marginally lower computational footprints, our solution delivers the optimal overall operational balance among high precision, low end-to-end latency ($\Delta t_{\text {e2e}}$), and resource efficiency in constrained edge computing environments.

## Introduction

The coming together of IoT devices, edge computing, and fast 5G/6G networks is pushing the use of communication models centered on data, with Named Data Networking (NDN) becoming a leading design for the future internet^1^. Unlike older models focused on network hosts, NDN shifts communication to named data, offering key benefits like efficient data storage within the network, built-in content verification, and direct multicast abilities^2^. However, this change in architecture creates specific security weaknesses due to how NDN manages connection states. A major threat among these is the Interest Flooding Attack (IFA), where attackers overwhelm the system by sending massive amounts of fake data requests (Interest packets) to fill up the network’s processing tables (Pending Interest Table, or PIT).This eventually slows down performance and blocks legitimate users from accessing content^3,4^.

The effective mitigation of IFAs in operational environments confronts multiple fundamental challenges. Contemporary adversaries increasingly employ sophisticated flooding strategies that mimic legitimate flash-crowd behavior, thereby complicating the discrimination between malicious and benign traffic patterns^5^. Furthermore, NDN traffic exhibits inherent heterogeneity across network segments, routers proximate to content producers versus those serving edge consumers encounter substantially different traffic distributions, violating the independent and identically distributed (IID) assumptions underpinning conventional centralized learning approaches^6^.

Existing countermeasures demonstrate notable limitations: while threshold-based mechanisms offer computational efficiency, their static nature frequently misclassifies legitimate demand surges as attacks, resulting in unacceptable false positive rates^7^. Centralized machine learning systems improve the precision of detection methods, but they require a great deal of network communication and pose a risk to privacy by revealing sensitive raw traffic data^8,9^. Approaches using blockchain technology offer better accountability and verification, but they introduce significant delays and demand too much computing power to be practical for environments with limited resources^10^.

FL is a new and significant approach for securing distributed systems in IoT and edge computing environments. It allows multiple devices to work together to train a shared security model without needing to exchange their sensitive raw data^11^, inherently improving privacy and lowering communication costs^12^.However, using FL to stop IFA presents specific problems. Because network traffic patterns often vary greatly across different routers (non-IID data), the shared model can become inconsistent or less effective. Additionally, malicious participants might try to compromise the integrity of the overall security model by submitting harmful or adversarial updates^13^.

This paper introduces FL-IFAshield, a complete federated learning framework designed to adaptively counteract IFA within NDN systems. The main contributions of this work are as follows:

- We developed a dynamic thresholding mechanism that synergistically integrates Poisson traffic modeling with Exponential Moving Average (EMA) filtering. Our approach enables the robust discrimination between malicious flooding and legitimate traffic surges while minimizing false positives.
- We design an entropy-weighted aggregation scheme that prioritizes model updates based on their informational value, effectively addressing data heterogeneity across routers and enhancing global model stability and accuracy.
- We integrate specific techniques namely gradient sparsification to make communication more efficient, differential privacy to protect sensitive data statistics, and Byzantine-robust aggregation mechanisms (specifically, the Krum algorithm) to defend against malicious participants attempting to compromise the security model.
- We implemented and tested the FL-IFAshield system on a large-scale IoT testbed. Our comprehensive evaluation covered several key performance metrics: how accurately it detected threats, its rate of false alarms, how quickly it responded to attacks, and how many system resources it used compared to several existing baseline methods.

To clearly distinguish FL-IFAshield from prior federated or centralized machine learning implementations in NDN security, its novelty lies not in the isolated execution of its sub-components, but in its domain-specific mathematical coupling. Conventional FL networks suffer severe degradation under heterogeneous, non-IID network traffic, as vanilla aggregation algorithms bias global weights towards high-volume benign routers. FL-IFAshield fundamentally alters this paradigm by embedding name prefix entropy directly into the federated aggregation matrix, effectively transforming a generic machine learning technique into an asymmetric, security-aware defense system optimized specifically for stateful NDN architectures.

This paper uses six distinct sections to present our research: We begin in Section "Background and related work" by offering a comprehensive overview of existing strategies for tackling IFAs and exploring how federated learning is currently applied in the context of network security. We dive in Section "System architecture and design" into the core of our work, detailing the architectural foundation and the theoretical basis of our proposed FL-IFAshield framework. Section "Implementation and system operation" outlines our experimental approach, providing specifics on how the testbed was configured, the characteristics of the datasets we used, and the metrics we applied to evaluate performance. The results of this evaluation, along with a comparative analysis against established baseline methods, are presented in full in Section "Experimental evaluation and analysis". Finally, Section "Conclusion and future research directions" brings the manuscript to a close by summarizing our principal research contributions in this field.

## Background and related work

### Background on named data networking and interest flooding attacks

NDN basically restructures how the Internet operates by adopting a content-centric approach that concentrates communication around the data itself^14^. Rather than relying on traditional host-centric models, the system retrieves information using unique content names. This new restructure offers significant benefits, including more efficient data delivery through in-network caching, intrinsic security via built-in content verification, and effective data distribution using native multicast capabilities. The most important to this architecture is the PIT, a crucial mechanism that maintains a record of outstanding requests (known as Interest packets) to manage the stateful forwarding and effective delivery of content.

However, this very mechanism of stateful forwarding inadvertently introduces a major security vulnerability known as the IFA. In an IFA, attackers introduce a large number of fake or impossible-to-fulfill data requests (Interest packets), which systematically fill up the network’s processing tables (PIT entries) and block legitimate requests from being handled^15,16^. This type of exploitation introduces substantial operational challenges, notably leading to considerable reductions in data throughput, increased end-to-end packet delivery latency $\Delta t_{e2e}$, and the possible onset of complete denial-of-service scenarios^17^. These attack vectors are especially impactful in settings with limited inherent network capacity, such as within Internet of Things (IoT) and edge computing frameworks^2^.

**Fig. 1:**
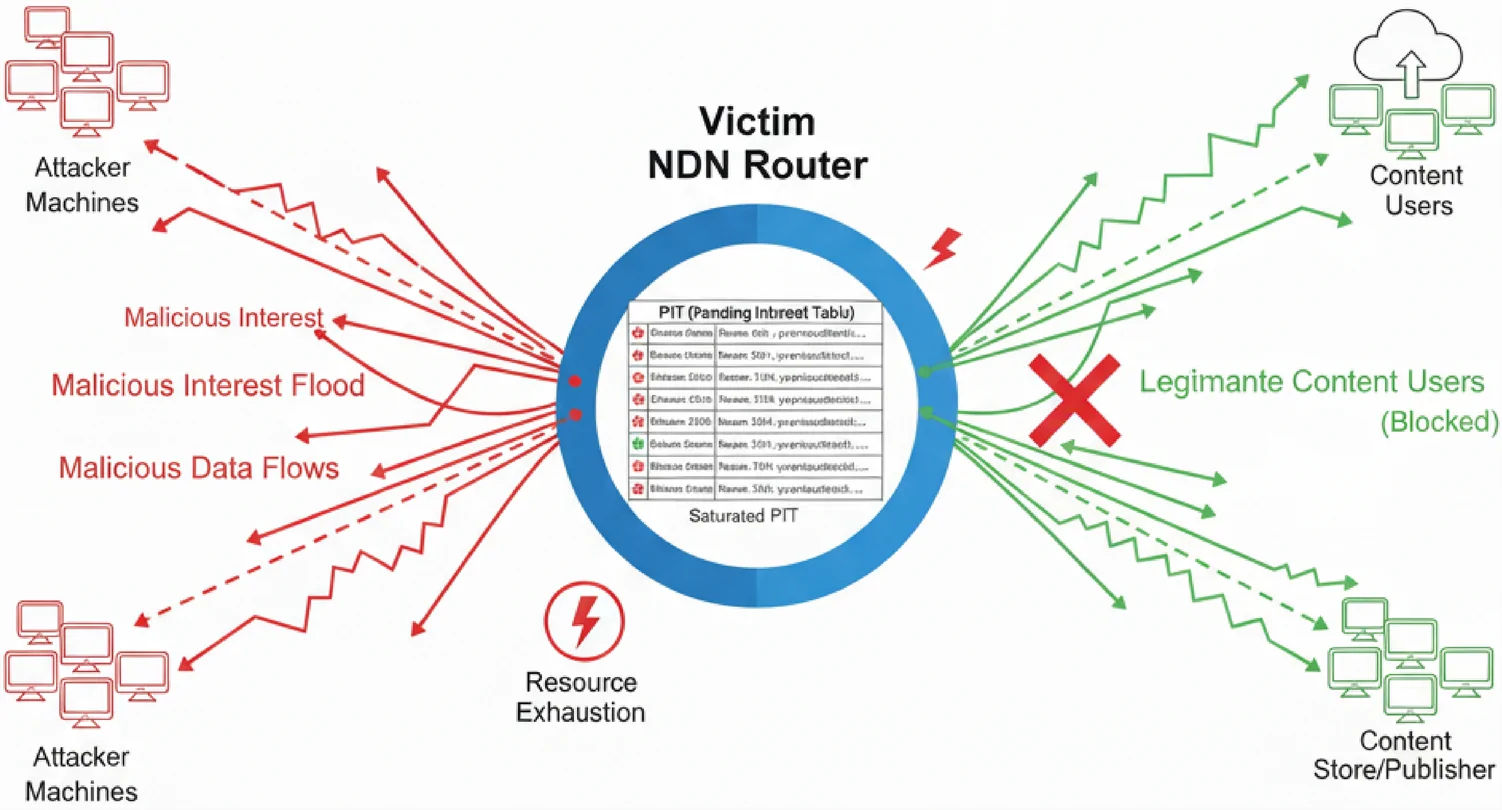
Mechanism of Interest Flooding Attack (IFA) in NDN. Malicious Interests saturate the Pending Interest Table (PIT), impeding legitimate request servicing and exhausting router resources.

Figure 1 visually details the operational methodology of the described attack (IFA). During standard network activity, authorized users submit requests (Interests) for data which content producers then fulfill. Attackers, conversely, simultaneously inundate the network infrastructure with fraudulent or unsolicited requests. These erroneous requests accumulate within the router’s PITs, which effectively prevents legitimate data packets from progressing and ultimately exhausts critical computational resources. This inherent vulnerability within the state management mechanism of NDN underscores the critical necessity for developing adaptive and scalable mitigation frameworks.

### Taxonomy of IFA mitigation approaches

The urgent requirement to effectively mitigate IFAs has driven extensive research efforts, leading to the development of a wide variety of defense mechanisms. These countermeasures are organized into four main categories:

1. Threshold-based detection mechanisms
2. Approaches utilizing machine and deep learning techniques
3. Defenses augmented by blockchain technology
4. Frameworks based on federated learning

Each of these approaches possesses its own unique strengths and inherent weaknesses, which we systematically compare and contrast in Table 1. Furthermore, recent reviews of the literature^1^ on NDN security and the use of federated learning for defending IoT systems confirm these trade-offs^18^. This reinforces the compelling need for developing hybrid, adaptive security solutions to ensure robust and effective protection in modern networks.

#### Threshold-based detection mechanisms

Detection mechanisms primarily rely on threshold-based defenses, which means they use straightforward monitoring rules (heuristics) to watch operational indicators^15,19,20^. These indicators include how full the PIT is, the ratio of requests to actual data, and how quickly those interests are satisfied. If any of these metrics cross a preset limit, the behavior is flagged as suspicious. The major benefit of this approach is its simplicity: the algorithms are easy to implement and require minimal computational power, making them ideal for network environments with limited resources, and they typically achieve the lowest absolute CPU footprint among all paradigms^5^. However, static thresholds present a significant drawback: they often struggle to differentiate between a malicious attack and a sudden, legitimate surge in traffic (like a popular website experiencing a flash crowd event). This frequently leads to a high number of false alarms (false positives)^5^. While newer techniques that adjust thresholds dynamically to accommodate varied traffic patterns^21^ offer a partial solution, applying these methods effectively across different types of network layouts (diverse network topologies) still poses a considerable challenge^22^.

#### Machine and deep learning approaches

To enhance the precision of detection systems, researchers are increasingly employing sophisticated approaches involving Machine Learning (ML) and Deep Learning (DL). These methodologies utilize complex statistical models to categorize network traffic patterns. A principal benefit of ML/DL is their inherent adaptability. Unsupervised learning techniques, which examine data without requiring prior labeling, often using methods such as entropy analysis or change-point detection^23^, are effective at identifying novel attack types. Conversely, supervised learning models, including Random Forests, Support Vector Machines (SVMs), and LSTMs (a specific type of neural network)^4,8^, have demonstrated resilience in identifying coordinated attacks (collusive IFAs) and issues originating within the PIT. Furthermore, hybrid systems that integrate these methods with theories like the Dempster-Shafer approach improve overall resilience against complex, mixed attack scenarios^24^. The field is advancing quickly as researchers introduce innovative techniques. Current research efforts involve incorporating reinforcement learning to optimize how the PIT is managed^25^, applying graph neural networks to understand interdependencies between network nodes^9^, and leveraging Software-Defined Networking (SDN) architectures for enhanced, centralized control^26^. Despite these ongoing advancements, Machine Learning (ML) and Deep Learning (DL) approaches still encounter practical hurdles: they demand significant processing capabilities, remain challenging to deploy directly on edge routers, and face difficulty adapting swiftly to highly dynamic shifts in network traffic conditions^27^.

#### Blockchain-assisted defense frameworks

Detection systems are increasingly leveraging Blockchain-Assisted Defense Frameworks by utilizing distributed ledger technologies (DLT).This approach provides several critical advantages: it creates logs that are nearly impossible to tamper with, establishes trust across a network without needing a central authority, and increases accountability among participants^10^. These features significantly enhance collaborative auditing and make it easier to trace events within distributed NDN environments. However, incorporating blockchain technology introduces a significant trade-off. The necessary ’consensus protocols’, the very mechanisms that make blockchains secure, require substantial computing power and communication bandwidth^28^. This inevitably slows down operations (increases latency) and limits how much the network can grow (scalability constraints). While recent efforts have focused on designing ’lightweight’ blockchains to lessen these impacts, their actual implementation in the low-resource NDN routers typically found in the field remains rare^2^.

#### Federated learning frameworks

Federated Learning (FL) has emerged as a promising new approach for mitigating In-Network Fast Attacks (IFAs) in a way that is both distributed and privacy-preserving^12^. The core benefit of FL is that it allows routers and IoT devices to collaboratively train a shared security model without needing to send sensitive, raw traffic data back to a central server. This design not only protects user privacy but also reduces the amount of network bandwidth used for communication^11,29^. Recent enhancements have improved this paradigm significantly, introducing techniques such as clustered FL architectures to handle uneven data distributions (non-IID traffic)^30^, entropy-based aggregation for better reliability, and the integration of differential privacy for stronger security guarantees^31^. Furthermore, advanced FL frameworks now use specialized methods that can tolerate malicious input (Byzantine-robust aggregation)^32^ and adaptive optimization to handle diverse data challenges^33^. Despite these significant advancements, FL systems still face persistent challenges. These include slow training convergence in varied network environments, ongoing vulnerability to sophisticated attacks like model poisoning, and the inherent communication overhead required to keep the locally trained models synchronized^6,13^.

**Table 1:** Taxonomy of IFA mitigation approaches in named data networking.

Category	Representative Works	Strengths	Limitations
Threshold-based Detection	^15,19–21^	*∙* Computational efficiency and lightweight implementation *∙* Straightforward deployment on constrained routers *∙* Minimal resource overhead	*∙* Limited adaptability of static thresholds *∙* Elevated false positives during flash crowds *∙* Poor scalability in dynamic traffic environments
Machine/Deep Learning	^4,8,9,23–26^	*∙* Superior detection accuracy across attack variants *∙* Capability to model complex traffic patterns *∙* Enhanced adaptability through RL, GNN, and SDN integration	*∙* Substantial computational and memory requirements *∙* Limited scalability on edge router platforms *∙* Vulnerability to concept drift in evolving traffic
Blockchain-assisted Defenses	^10,28^	*∙* Decentralized trust management and accountability *∙* Tamper-resistant audit logging *∙* Strong security guarantees through cryptographic verification	*∙* Significant consensus protocol overhead *∙* Increased operational latency *∙* Scalability challenges in IoT/NDN environments
Federated Learning Frameworks	^11,6,29–31,33^	*∙* Privacy-preserving collaborative training *∙* Adaptability to heterogeneous and non-IID data distributions *∙* Improved generalization through distributed learning	*∙* Sensitivity to data heterogeneity across participants *∙* Communication overhead from model synchronization *∙* Vulnerability to poisoning and Byzantine attacks

As summarized in Table 1, current security approaches individually fail to meet all the interdependent requirements for effective defense: scalability, adaptability, privacy preservation, and robustness. For example, while threshold-based defenses are efficient, they lack the necessary flexibility to handle diverse traffic. ML/DL techniques offer high accuracy but are often too complex for practical deployment in resource-limited environments. While blockchain mechanisms guarantee robust accountability, they come at the cost of introducing prohibitive operational delays (latency). Similarly, FL frameworks excel at preserving data privacy but encounter challenges regarding data variability (heterogeneity) and the significant communication overhead required for model synchronization. Comprehensive studies^1,18^ confirm these fundamental trade-offs, leading to the conclusion that integrated, hybrid solutions are necessary. Motivated by these findings, our proposed FL-IFAshield framework advances the current state-of-the-art by combining the best aspects of both adaptive thresholding and lightweight federated learning. This synergy results in a robust, privacy-preserving, and practical solution tailored specifically for resource-constrained NDN environments.

Our system directly addresses the core challenges of federated learning in distributed NDN environments. To overcome sensitivity to data heterogeneity, we employ an entropy-weighted aggregation scheme that prioritizes model updates from routers experiencing diverse, informative traffic, ensuring stability and accuracy under non-IID conditions. Communication overhead is minimized through gradient sparsification, transmitting only the most significant updates, and optimized local feature extraction, which drastically reduces payload size without compromising detection quality. Finally, vulnerability to poisoning and Byzantine attacks is mitigated by integrating the Krum aggregation algorithm, which filters out malicious updates by selecting those most consistent with their peers, thereby preserving the integrity of the global model. Together, these mechanisms enable robust, efficient, and secure collaborative learning tailored for resource-constrained edge routers.

While contemporary FL designs acknowledge these generic limitations, prior NDN implementations fail to address the operational intersection of non-IID router placement and resource constraints. FL-IFAshield bridges this gap by replacing computationally heavy cryptographic alignment layers with lightweight, traffic-driven information-entropy constraints, reducing edge router overhead below 9% CPU utilization while actively filtering Byzantine adversaries, Table 2.

**Table 2:** Architectural and theoretical differentiation matrix.

Dimension/Metric	Prior ML/DL Systems	Blockchain Systems	FL-IFAshield (Ours)
Non-IID Aware	No	Yes	Yes (Entropy-Weighted)
Privacy Guarantees	Low	Moderate	High (DP + Krum)
Mitigation Feedback Loop	No	No	Yes (Closed-Loop)
Edge Router CPU Footprint	High (*> 12%*)	Prohibitive (*> 17%*)	Minimal (*< 9%*)

## System architecture and design

This section presents the architectural design of FL-IFAshield, a privacy-preserving adaptive defense framework against IFAs in NDN.The proposed architecture addresses critical limitations of existing approaches, including privacy vulnerabilities in centralized learning systems^29^, the inflexibility of static threshold mechanisms^19^, and the prohibitive overhead of cryptographic and blockchain-based solutions^28^.

### Architectural overview

**Fig. 2:**
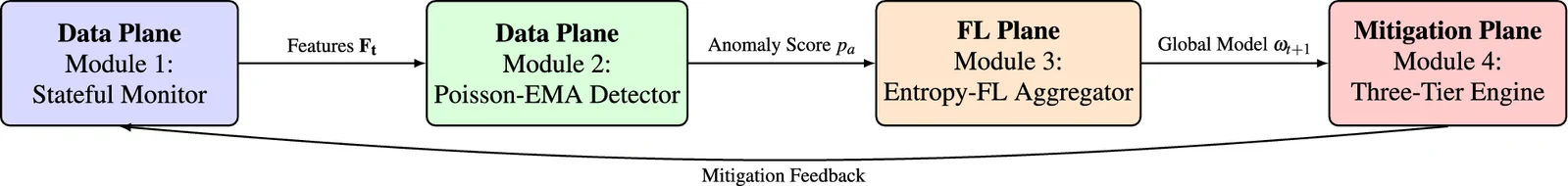
Three-plane, four-module architecture of the FL-IFAshield framework. Data flow and feedback mechanisms enable continuous adaptation to evolving threats.

The architecture of FL-IFAshield uses a hierarchical multi-plane design, effectively structured into four distinct modules that work together seamlessly.The entire system operates on the principle of “collaborative intelligence”: individual routers contribute to a collective threat assessment effort without ever exposing their sensitive, raw traffic data. This design guarantees two key outcomes: the robust detection of sophisticated attacks and full compliance with data protection regulations^34^.It also specifically addresses the practical resource constraints common in edge computing environments. Figure 2 provides a high-level visual overview of this architecture, illustrating how the four core modules interact within a continuous cycle that includes monitoring, detection, collaborative learning, and threat mitigation.

### Module 1: Stateful monitor

The Stateful Monitor operates as a continuous, local surveillance component on each NDN router, performing real-time observation of PIT states and incoming Interest traffic characteristics. All processing occurs locally to ensure no raw packet data or PIT entries are externally transmitted, thereby preserving user privacy and minimizing bandwidth consumption^21^.The module ingests a stream of input parameters, including real-time PIT events (insertions and deletions via satisfaction or timeout), the hierarchical name prefixes of incoming Interest packets, and their precise arrival timestamps. From these inputs, it extracts a set of condensed statistical feature vectors, $\mathbf {F_t}$, which characterize router traffic patterns; these features are selected for their demonstrated efficacy in discriminating between legitimate traffic and IFA patterns^23,35^.

Its core functionality is twofold. First, it conducts continuous PIT State Analysis, monitoring PIT occupancy levels to use rapid PIT saturation with unsatisfied entries as a primary indicator of potential attacks^15^. The second step involves Traffic Feature Extraction, where the system computes several key metrics from the network flow. A critical calculation here is the Name Prefix Entropy ($\text {H}_{t}$), which scientifically measures the level of randomness and diversity found in the requested names. When this entropy value suddenly spikes, it usually signals an attack scenario that uses randomly generated names to overwhelm the system^16^. This metric is precisely defined by the accompanying equation.

1 $$\begin{aligned} H_t = -\sum _{i=1}^{N} p_i \log _2 p_i \end{aligned}$$

where $p_i$ represents the probability of the *i*-th name prefix within the current observation window; elevated values correspond to uniformly distributed requests (an attack characteristic), while reduced entropy indicates concentrated, popular content. The module also tracks the Interest Satisfaction Ratio (ISR), where declining values suggest an increasing rate of requests for non-existent content, a fundamental IFA signature^17^. Finally, it analyzes Interest Inter-arrival Statistics, as attack traffic typically exhibits distinct burstiness patterns, such as sustained high rates, compared to legitimate human or application-driven traffic^5^. The final output of the module is a continuous stream of these condensed statistical feature vectors alongside PIT health metrics and operational status indicators.

### Module 2: Poisson-EMA detector

**Role and Functionality:** This system acts as a smart network guard, performing the first, automatic check-up by watching for major, unusual spikes away from the network’s normal, expected flow. Its main breakthrough is using flexible, data-driven limits (adaptive thresholds). These limits are intelligent enough to tell the difference between a hostile attack (like malicious flooding intended to crash the system) and a sudden, legitimate rush of users (like a “flash crowd” hitting a popular website). By making this crucial distinction, the system drastically cuts down on false alarms, a common headache in older security tools^19^.

This component receives its input parameters from the Stateful Monitor, consisting of the feature vector stream $\mathbf {F_t}$ and specifically the interest count $\mathbf {X_t}$. It also utilizes a continuously refined baseline that establishes normal traffic characteristics. The central operation is performed by the Poisson-EMA Methodology, a dual-process technique for determining dynamic detection thresholds. This methodology integrates Poisson modeling to characterize discrete events with Exponential Moving Average (EMA) smoothing to ensure temporal stability in the results^5,20^.

The algorithm is executed in three principal steps. It begins with Poisson Modeling, a process where the arrival of legitimate network data units, or Interest packets, is mathematically treated as a Poisson process over brief time periods. This formalization accurately characterizes these arrivals as randomly occurring, independent events^5^. The module then proceeds to determine the average arrival rate $\lambda _t$). This is achieved by calculating the Maximum Likelihood Estimator (MLE) for the Poisson distribution’s rate parameter, utilizing the observed packet counts within a preceding time window of size *W*.

2 $$\begin{aligned} \lambda _{t} = \frac{1}{W} \sum _{i=t-W+1}^{t} X_i \end{aligned}$$

Second, Exponential Smoothing is employed to mitigate false alarms from transient spikes and to facilitate gradual adaptation to long-term trends. The estimated rate is processed through an Exponential Moving Average (EMA) with a smoothing factor *α* (*0< α < 1*) that governs the weighting of recent observations, yielding a stabilized baseline $\mu _t$:

3 $$\begin{aligned} \mu _{t} = \alpha \cdot X_{t} + (1-\alpha ) \cdot \mu _{t-1} \end{aligned}$$

The final step is the Dynamic Threshold Calculation, which is established by integrating the smoothed baseline (the output of the EMA process) with a statistical confidence bound. The detection threshold is established by aggregating the estimated mean of the packet counts ($\mu _{t}$) with a specific multiple of the associated standard deviation. This method capitalizes on a distinctive property of the Poisson distribution, namely the equivalence between its expected value (mean) and its variability measure (variance). Consequently, the standard deviation is defined as the square root of the mean. The ultimate detection threshold $\tau _t$ is then computed by adding this standard deviation term, scaled by a sensitivity multiplier *k*. This multiplier controls the tolerance for deviations (often *k*=3 is used, corresponding to a *3σ* confidence level in many statistical models)

4 $$\begin{aligned} \tau _{t} = \lambda _{t} + k \sqrt{\mu _{t}} \end{aligned}$$

The selection of the core parameters within the Poisson-EMA module—namely the sliding window size *W*, the smoothing factor *α*, and the sensitivity multiplier *k*—directly governs the equilibrium between detection sensitivity and the false positive rate (FPR). To optimize these values, an automated grid search sweep was conducted over our physical deployment fabric.

The sliding window size *W* designates the temporal horizon utilized to estimate the baseline Poisson arrival rate $\lambda _{t}$ (Eq. 2). A restrictive window size induces high variance due to minor, transient packet fluctuations, whereas an excessively expanded window delays responsiveness to rapid-onset attacks. Empirical optimization identified *W = 30* epochs as the optimal balance, ensuring rapid detection convergence while preserving macro-level traffic context.

The exponential moving average factor *α* controls the memory decay rate, balancing immediate rate variations against historical traffic trends (Eq. 3). Setting *α = 0.2* ensures that the framework filters out high-frequency network jitter and transient micro-bursts, yet adapts smoothly to long-term diurnal traffic shifts. Table 3 highlights the importance of paramerer selection within the Poisson-EMA module.

Finally, the multiplier *k* scales the statistical confidence bound attached to the dynamic threshold $\tau _{t}$ (Eq. 4). Selecting *k = 3* map to a standard *3σ* statistical confidence domain. This boundary restricts the empirical false positive rate to a strictly bounded *2.63%* under normal operation and flash crowds, while securing high discriminative capacity (*93.1%* overall F1-score) under coordinated, multi-prefix Interest Flooding Attacks.

This adaptive threshold automatically adjusts to prevailing traffic conditions, remaining sensitive during low-traffic periods while elevating to prevent false positives during legitimate high-traffic intervals. Any incoming rate exceeding this statistically derived boundary triggers an anomaly classification. The outputs of this module are a local anomaly score $p_a^{(local)}$, which quantifies the router’s confidence in an attack detection, and binary alert signals for high-confidence local detections.

**Table 3:** Poisson-EMA hyperparameter selection and optimization matrix.

Parameter	Operational Value	Primary Optimization Vector	Boundary Trade-off
*W*	30 Epochs	Historical baseline calculation ($\lambda _t$)	Low values cause baseline jitter; high values slow detection latency.
*α*	0.2	Exponential temporal smoothing ($\mu _t$)	Low values cause sluggish adaptation; high values trigger false alarms.
*k*	3.0	Statistical confidence boundary ($\tau _t$)	Low values increase FPR; high values cause false negatives (missed attacks).

### Module 3: Entropy-FL aggregator

**Role and Functionality:** This module facilitates collaborative learning across the router network, enabling knowledge sharing without privacy compromise. It specifically addresses two fundamental challenges in distributed network learning: non-uniform traffic patterns across routers (non-IID data)^21^ and potential compromise of participating nodes (Byzantine participants)^32^.

The module’s **input parameters** include the encrypted model updates (gradients) from all participating routers, along with metadata such as their local dataset sizes $|D_i|$ and entropy-derived confidence scores $c_i$. Its **core operations** involve orchestrating periodic training rounds to enhance a shared global detection model, extending the foundational Federated Averaging (FedAvg) algorithm^36^ with critical improvements for security and performance. The process begins with Entropy-Weighted Aggregation. Recognizing that conventional FedAvg, which prioritizes updates based solely on dataset size, is inadequate for non-IID network data, this module incorporates a dual-weighting mechanism. Since routers encountering diverse attack traffic (high entropy) provide more informative learning signals than those processing repetitive legitimate traffic^30^, the aggregation weights updates using both dataset size and confidence:

5 $$\begin{aligned} w_i = \frac{c_i \cdot |D_i|}{\sum _{j=1}^{N} c_j |D_j|} \end{aligned}$$

This ensures the global model is biased towards contributions from routers with both substantial data volumes and informative, diverse data. To optimize communication and provide privacy, the module employs **Gradient Sparsification and Differential Privacy**. Routers transmit only the top-*k* gradients by magnitude^36^, and calibrated Gaussian noise $\mathscr {N}(0, \sigma ^2)$ is injected into these updates. This differential privacy technique^37^ provides mathematically rigorous guarantees, impeding the inference of raw training data from updates and protecting against gradient inversion attacks^38^:

6 $$\begin{aligned} \tilde{G}^{(i)} = \text {TopK}\left( G^{(i)}, k\right) + \mathscr {N}(0, \sigma ^2) \end{aligned}$$

While the injection of Gaussian noise $\mathscr {N}(0,\sigma ^{2})$ provides mathematically rigorous differential privacy guarantees ($\epsilon , \delta$) against gradient inversion attacks, it inherently introduces a minor trade-off in empirical model accuracy. Through hyperparameter optimization during our testbed deployment, the noise scale *σ* was calibrated to maximize privacy preservation while maintaining the global F1-score drop within a negligible $\le 1.5\%$ margin compared to non-private baselines.

The noise scale *σ* is calibrated to achieve a specified privacy budget $(\epsilon , \delta )$, balancing privacy with model utility. Finally, **Byzantine-Robust Aggregation** is applied to withstand malicious participants. Before averaging, the module employs the Krum algorithm^32^, which identifies the model updates most consistent with their neighbors, effectively filtering out outliers from Byzantine nodes:

7 $$\begin{aligned} \text {Krum}(\{G^{(i)}\}) = \arg \min _{G^{(i)}} \sum _{j \rightarrow i} ||G^{(i)} - G^{(j)}||^2 \end{aligned}$$

For each candidate update $G^{(i)}$, Krum computes the sum of squared distances to its nearest neighbors and selects the one with the minimal aggregate distance as the most trustworthy consensus. The final **output** of this process is an enhanced, robust global detection model that is distributed back to all participating routers.

It is worth noting that the integration of the Krum algorithm introduces a theoretical Byzantine boundary where the framework successfully maintains integrity provided that the number of compromised or malicious routers *f* satisfies the condition *2f + 2 < N*, where *N* represents the total number of participating nodes. In operational scenarios where edge compromise exceeds this threshold, a fallback consensus layer or localized trust scoring is triggered to dynamically isolate highly divergent clusters, preserving global model convergence.

### Module 4: Three-tier mitigation engine

**Role and Functionality:** This module executes defensive actions based on detection confidence scores ($p_a$). Its design embodies the principle of *proportional response*^39^, applying minimally sufficient mitigation to neutralize threats while minimizing impact on legitimate traffic^22,40^.

The **Mitigation Engine** operates based on a set of critical input parameters: the final anomaly probability $p_a$, which integrates both local and global model assessments; the current PIT occupancy level; and feedback regarding the effectiveness of any active mitigation measures. Its **core operations** are implemented as a state machine that executes an escalating response strategy, where defensive measures intensify in proportion to the increasing certainty of an attack. The engine’s response is structured across three distinct tiers. In **Tier 1: Rate Limiting**, triggered at a medium confidence level ($p_a \ge T_{alert}$), the engine implements token bucket filters on suspicious prefixes^20^.

This action gently throttles request rates to decelerate the flood and stabilize the PIT without causing a complete service denial, thereby minimizing the impact of potential false positives. If rate limiting proves insufficient and the anomaly probability reaches a high confidence level ($p_a \ge T_{mitigate}$), the engine escalates to **Tier 2: PIT Partitioning**. Here, it employs logical attack isolation using techniques inspired by Stateful Interest Casting (SIC)^40^, allocating quarantined sections of the PIT for suspicious traffic while reserving guaranteed capacity exclusively for verified legitimate traffic to ensure service continuity.

For confirmed malicious prefixes at a very high confidence level ($p_a \ge T_{critical}$), the engine activates **Tier 3: Cryptographic Blacklisting**. It issues cryptographically signed blacklist entries^10^ to prevent forgery and enable secure, coordinated network-wide defense. These blacklists implement temporary durations with exponential backoff (e.g., 30 s, 60 s, 120 s) to allow for automatic expiration and prevent permanent denial-of-service from potential misidentification. A continuous feedback loop monitors PIT occupancy levels, and persistently critical levels following mitigation automatically trigger escalation to the next defensive tier, forming an adaptive closed-loop control system. The **outputs** of this engine are direct commands to the router’s forwarding plane for rate-limiting, PIT management, or filtering operations, as well as signed blacklist messages for distributed network-wide implementation.

### System workflow integration

**Fig. 3:**
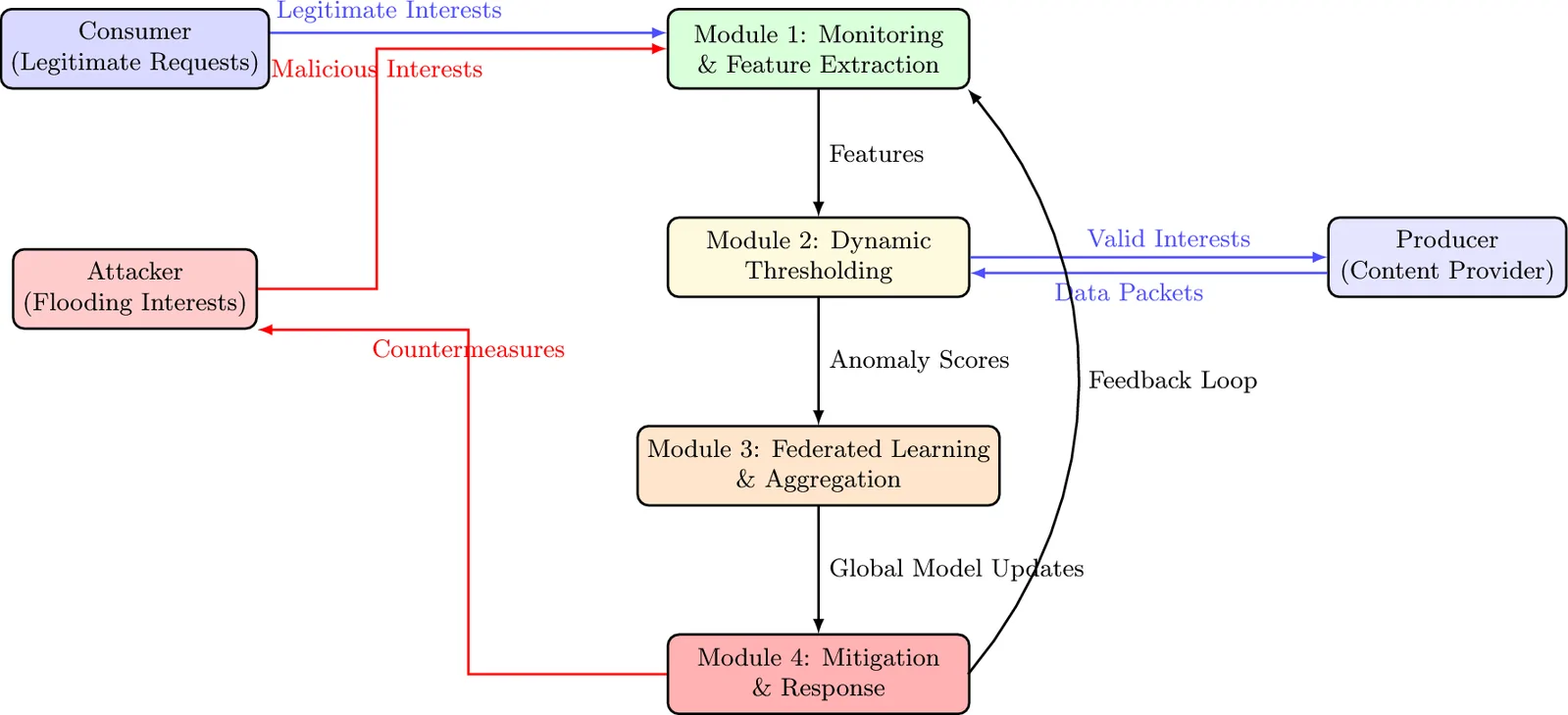
Comprehensive workflow of the IFA mitigation framework. Legitimate and malicious interests undergo monitoring and feature extraction, with subsequent dynamic thresholding and federated learning collaboration for robust detection. Valid interests proceed to producers while malicious traffic triggers mitigation, with continuous adaptation via feedback mechanisms.

Figure 3 illustrates the comprehensive operational workflow. Traffic initially enters the *Monitoring and Feature Extraction* module, where PIT states are collected and key statistical indicators derived. These features proceed to the *Dynamic Thresholding* module, where adaptive thresholds identify deviations from normal traffic patterns. Suspicious instances trigger collaborative analysis through the *Federated Learning and Model Aggregation* module, where local models train and securely combine into robust global models. Upon attack confirmation, the *Mitigation and Response Engine* enforces countermeasures including rate limiting, PIT partitioning, or blacklist isolation. Mitigation outputs feed back into monitoring, establishing a closed-loop system that continuously adapts to evolving IFAs. This integrated modular approach enables precise detection, rapid response, and resilient protection in practical NDN deployments.

**Table 4:** FL-IFAshield framework: component summary and functional characteristics.

Module	Primary Function	Core Technique(s)	Key Innovation	Addressed Challenge
Stateful Monitor	Real-time feature extraction	PIT analysis, Prefix Entropy ($H_t$), ISR	On-device processing; Privacy-by-design	Privacy violation; Resource overhead
Poisson-EMA Detector	Adaptive anomaly detection	Poisson MLE, Exponential Moving Average (EMA)	Dynamic threshold ($\tau _t$); Hybrid statistical modeling	Adaptability gap; False positives during flash crowds
Entropy-FL Aggregator	Collaborative model training	Entropy-weighted aggregation, Gradient sparsification, Krum aggregation	Handles non-IID data; Byzantine robust; Privacy-preserving	Non-IID data; Model poisoning; Privacy leakage
Three-Tier Mitigation Engine	Proportional attack response	Rate limiting, PIT partitioning, Cryptographic blacklisting	Graduated response; Feedback loop; Minimal collateral damage	Latency constraints; Service continuity; Over-mitigation

Table 4 provides a consolidated overview of the FL-IFAshield framework architecture, delineating how each component addresses fundamental IFA mitigation challenges. The table demonstrates the logical progression from privacy-preserving feature extraction in the Stateful Monitor through adaptive local detection via the Poisson-EMA Detector. It further illustrates how the Entropy-FL Aggregator facilitates collaborative learning while ensuring robustness against non-IID data and Byzantine attacks, culminating in the Three-Tier Mitigation Engine’s proportional response strategy. This systematic design resolves the critical trilemma of privacy preservation, adaptability, and resource efficiency that impedes existing solutions.

## Implementation and system operation

This section delineates the practical implementation of the FL-IFAshield framework, which is integrated as a software module within the Named Data Networking Forwarding Daemon (NFD). We detail the deployment considerations for resource-constrained environments, the specific implementation of each core module, the synergistic workflow that orchestrates their interaction, and the data flow that enables real-time, privacy-preserving mitigation of Interest Flooding Attacks (IFAs).

### Resource-constrained deployment

The FL-IFAshield architecture is designed from the ground up for deployment on resource-constrained edge routers. To quantify its feasibility, Table 5 summarizes the estimated computational overhead for each module when operating on a commodity 4-core ARMv8 device, representative of hardware such as a Raspberry Pi 4. These estimates are derived from the computational complexity of the underlying algorithms and are informed by prior benchmarking studies conducted in *ndnSIM*^39^, PIT management research^22^, and federated learning analyses on IoT devices^21,36^. The results demonstrate the framework’s potential for carrier-grade scalability under realistic hardware constraints.

**Key optimizations** that underpin this efficiency include:

- **Memory Pooling:** Pre-allocated ring buffers for PIT statistics eliminate dynamic memory allocation overhead during traffic surges, ensuring deterministic performance.
- **Model Quantization:** The lightweight convolutional and federated learning models employ FP16 quantization and INT8 activations, reducing the memory footprint by approximately 60% without significant degradation in detection accuracy.
- **Lock-Free Data Structures:** The Stateful Monitor utilizes lock-free ring buffers for exchanging metrics between the data plane and detection threads, thereby minimizing context-switching delays and enhancing real-time responsiveness.

Collectively, these optimizations enable FL-IFAshield to operate within the stringent resource budgets of commodity ARMv8 hardware while delivering adaptive, federated protection against IFAs.

**Table 5:** Estimated resource utilization of FL-IFAshield modules on a 4-core ARMv8 router.

Component	CPU Usage (%)	Memory (MB)	Local Execution Delay (ms)
Stateful Monitor	3.2	45	*<1.0*
Poisson-EMA Detector	2.1	22	0.8
Entropy-FL Aggregator	2.1	68	–
Mitigation Engine	1.5	34	0.3
**Total**	**8.9**	**169**	*<2.1*

1. The sum of the Stateful Monitor and Poisson-EMA Detector delays (*≤*1.8 ms) constitutes the local component of Detection Latency ($\Delta t_{\text {det}}$).

2. The Mitigation Engine execution delay (0.3 ms) represents the core operational footprint of Mitigation Latency ($\Delta t_{\text {mit}}$).

### Module implementation and algorithms

#### Module 1: Stateful monitor

The Stateful Monitor operates as a high-priority thread within the NFD’s forwarding pipeline. It is triggered by key forwarding plane events: Interest arrival, Data packet satisfaction, and PIT entry timeout. Its continuous operation, which involves feature extraction and vector packaging, is formalized in Algorithm 1.

**Algorithm 1:**
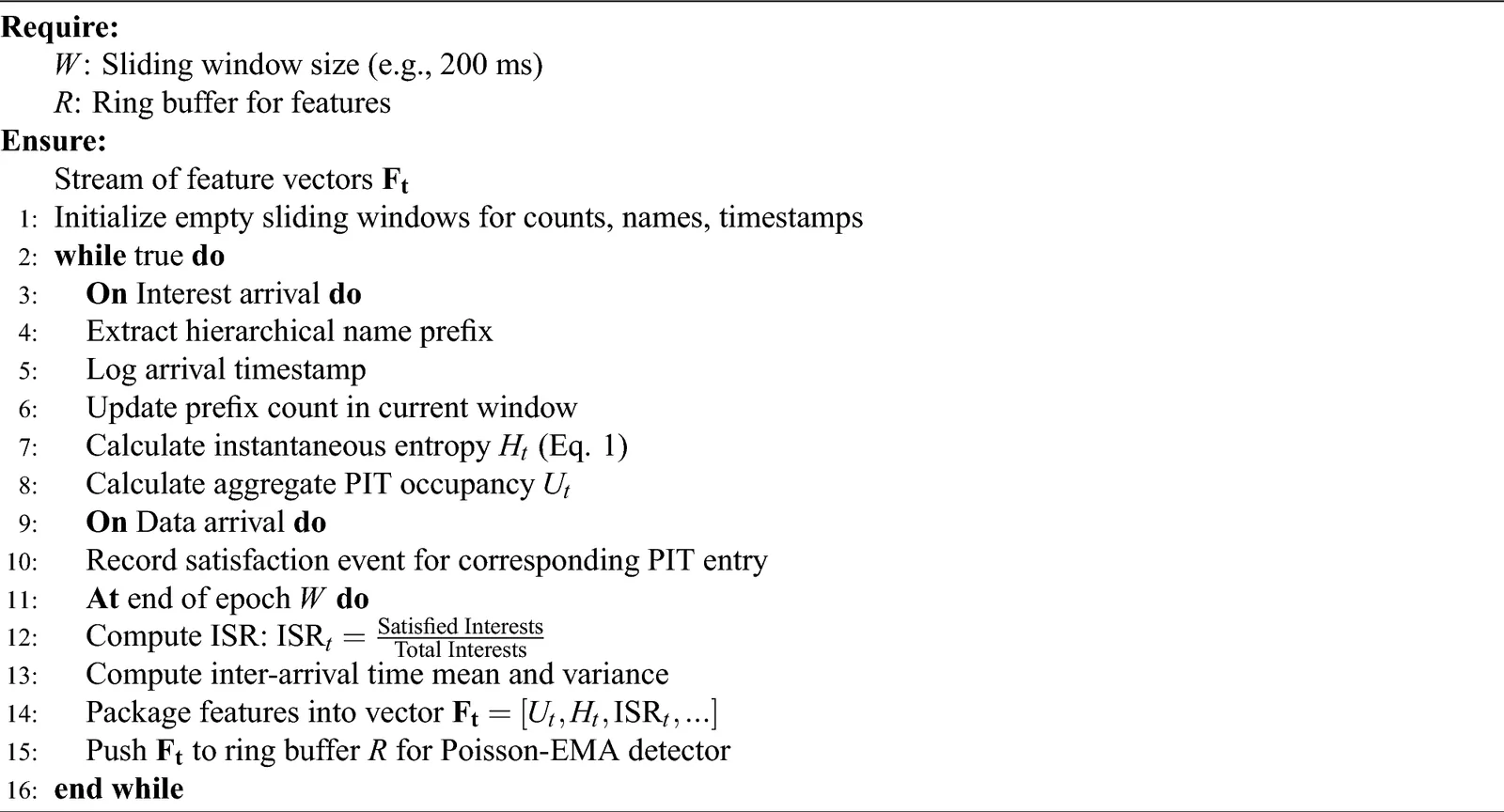
Stateful monitor operation

#### Module 2: Poisson-EMA detector

This module consumes the stream of feature vectors from the ring buffer. Algorithm 2 encapsulates the dynamic thresholding logic, which synergistically combines Poisson modeling for baseline estimation with Exponential Moving Average (EMA) smoothing for stability, as detailed in Section.

**Algorithm 2:**
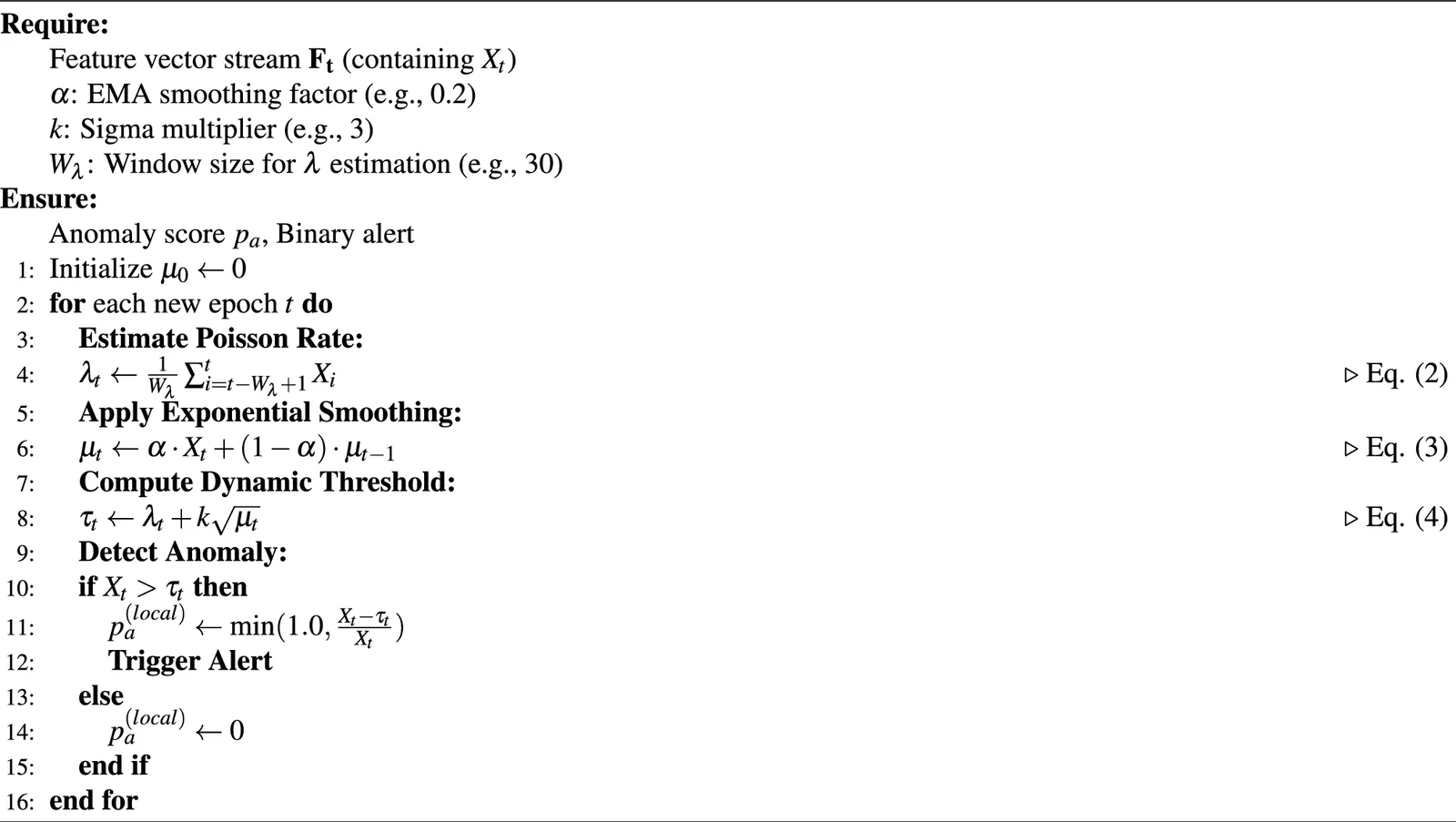
Poisson-EMA dynamic thresholding

#### Module 3: Entropy-FL aggregator

The federated learning service is implemented as a separate microservice that communicates with routers via secured NDN Interest and Data packets. The core aggregation process, which incorporates entropy-weighted averaging, gradient sparsification, differential privacy, and Byzantine-robust selection, is detailed in Algorithm 3.

**Algorithm 3:**
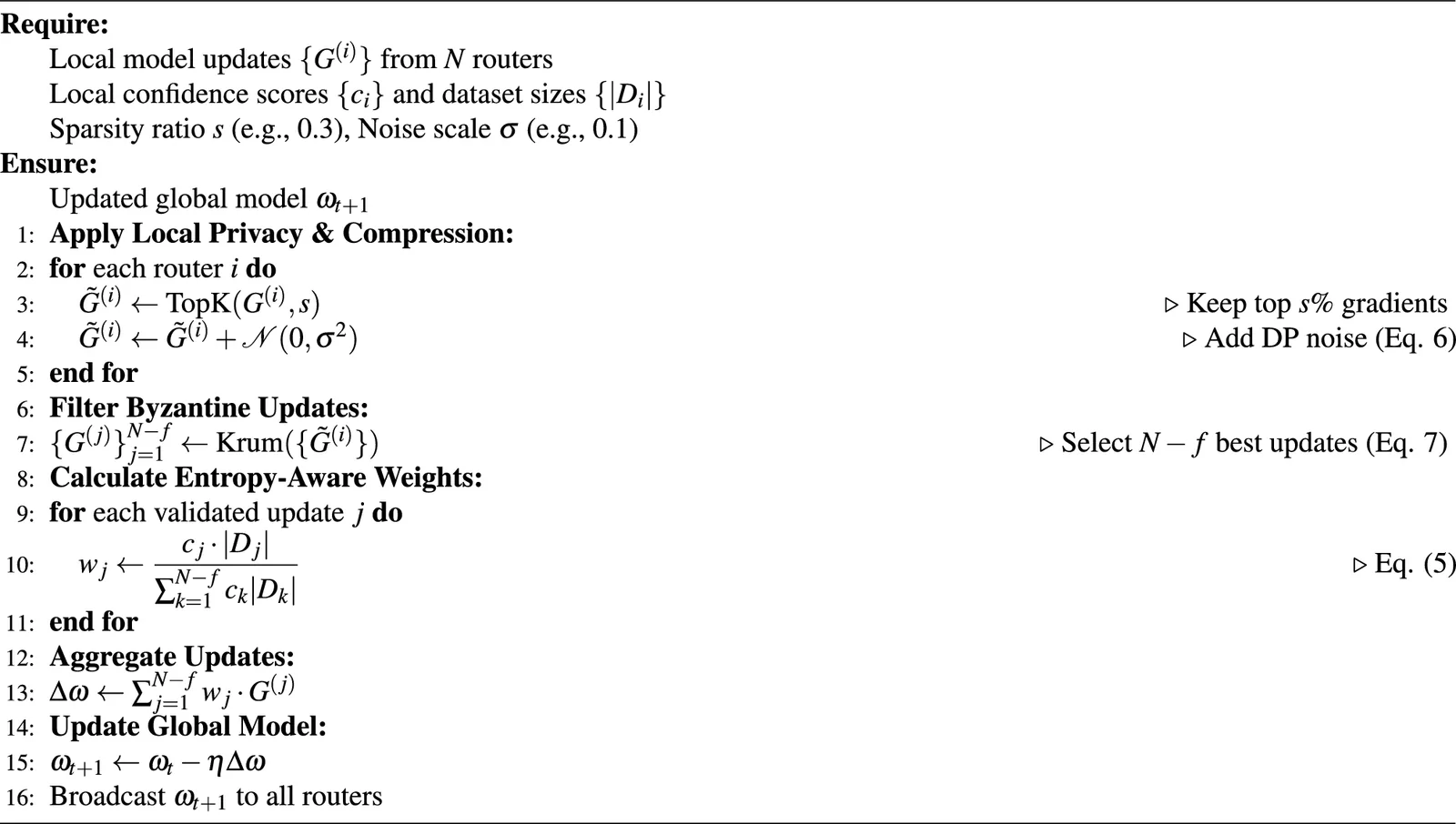
Entropy-weighted secure aggregation

#### Module 4: Three-tier mitigation engine

The mitigation engine is implemented as a state machine that interfaces directly with the NFD’s forwarding plane. Its decision logic, which enforces a proportional response strategy based on anomaly confidence and PIT state, is formalized in Algorithm 4.

**Algorithm 4:**
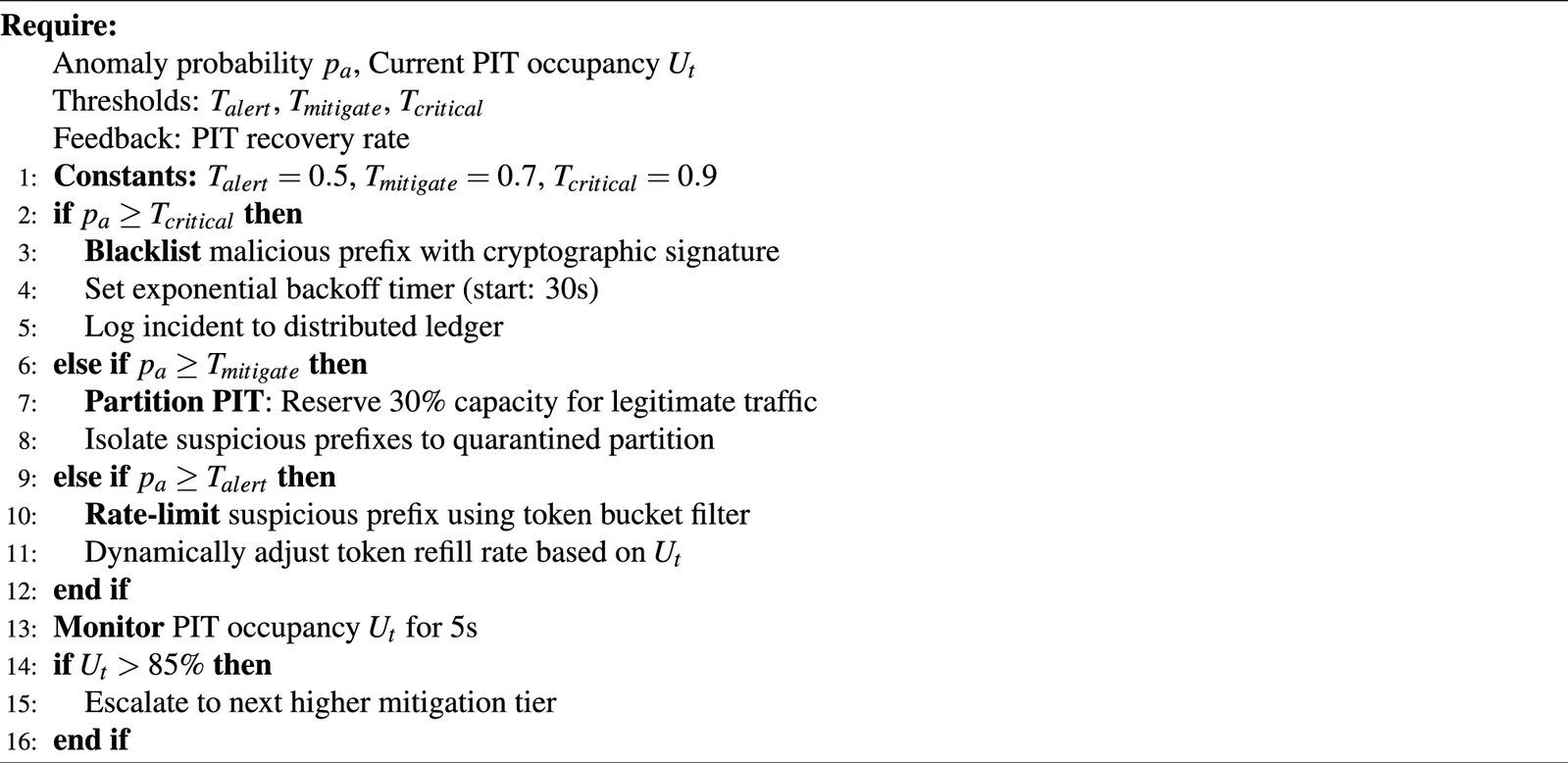
Three-tier mitigation state machine

### System workflow and data flow

**Fig. 4:**
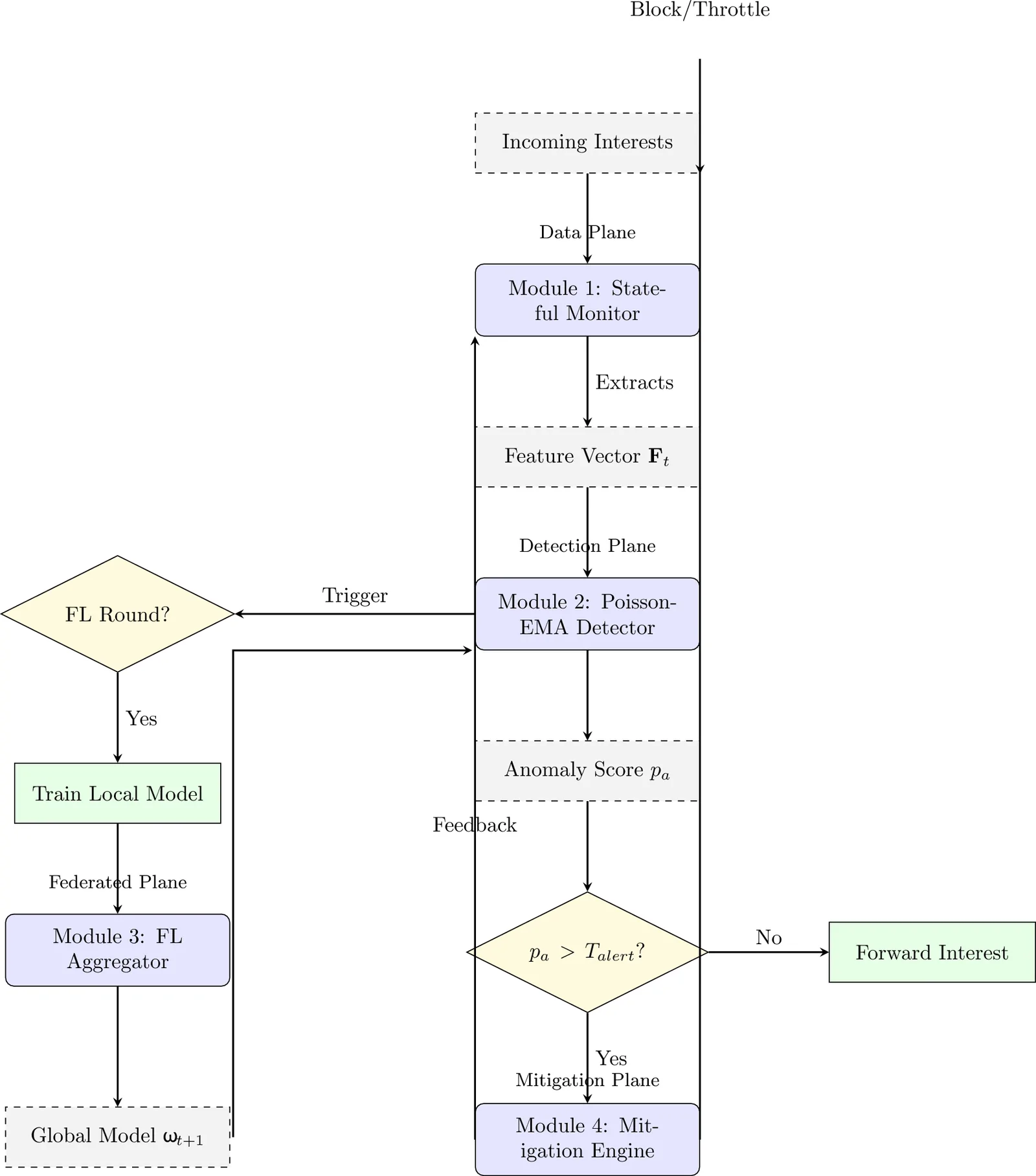
Optimized FL-IFAshield workflow with enlarged nodes and text, resized to page width for IEEE readability.

The operational workflow of FL-IFAshield, illustrated in Fig. 4, demonstrates the sequential and parallel interaction of its modules to process traffic and mitigate attacks in real-time. The process can be described in the following steps:

**Table 6:** Feature Vector $\mathbf {F_t}$ with formulas, behavioral indicators, and usage. Novel features are in italics.

Feature	Formula	Behavioral Indicator	Usage
$O_{pit}$	$\frac{\text {Occupied}}{\hbox {PIT size}}$	PIT saturation	Early anomaly (M1, M2)
$R_{isr}$	$\frac{\text {Satisfied}}{\hbox {Total Interests}}$	Service success ratio	Benign vs. attack (M2)
$R_{dir}$	$\frac{\text {Data}}{\hbox {Interests}}$	Response efficiency	Validate efficiency (M2, M3)
$R_{ret}$	$\frac{\text {Retransmissions}}{\hbox {Interests}}$	Failed request persistence	Repeated failures (M1, M2)
$H_{prefix}$	$-\sum p_i \log p_i$	Prefix randomness	FL weighting, anomaly (M3)
$\sigma _{iat}$	$\textrm{Var}(\Delta t)$	*Traffic burstiness*	*Bursty traffic (M2)*
$B_{req}$	*Windowed burst score*	*Short-term surges*	*Flood detection (M1, M2)*
$EMA_{rate}$	$\alpha x_t+(1-\alpha )EMA_{t-1}$	*Smoothed traffic trend*	*Adaptive thresholds (M2)*

**Fig. 5:**
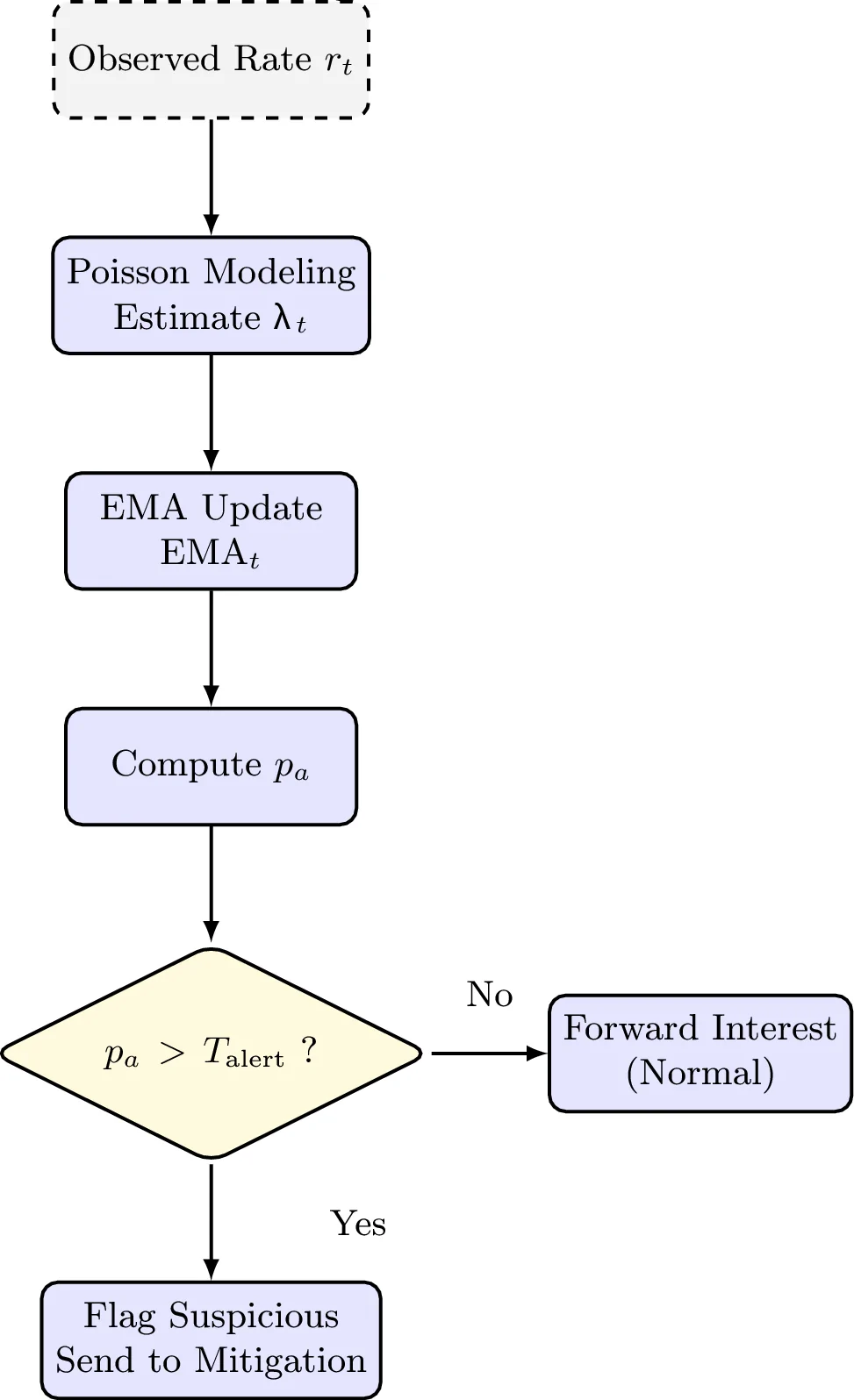
Workflow of the poisson-EMA anomaly detector (Module 2).

1. **Incoming Interests:** Both legitimate consumer requests and malicious flooding packets enter the router.
2. **Module 1 – Stateful Monitoring:** The system inspects PIT occupancy, satisfaction ratio, and prefix distribution, generating a statistical *feature vector* $\mathbf {F_t}$ that concisely summarizes traffic behavior. **Feature Vector Composition:** The feature vector $\mathbf {F_t}$ serves as the core representation of traffic behavior, enabling efficient local detection and collaborative learning without transmitting raw data. Formally, the vector is defined as:
  $$\textbf{F}_t = [O_{\text {pit}}, R_{\text {isr}}, R_{\text {dir}}, R_{\text {ret}}, H_{\text {prefix}}, \sigma _{\text {iat}}, B_{\text {req}}, \text {EMA}_{\text {rate}}]$$
  **Novelty Statement:** Beyond traditional IFA detection metrics^27^, our system introduces three novel features to enhance discriminative power: (i) $\sigma _{iat}$ captures temporal burstiness^27^, (ii) $B_{req}$ detects short-term flood surges^23^, and (iii) $EMA_{rate}$ leverages smoothed trends to better differentiate flash crowds from attacks^41^. This integration of dynamic, burst-sensitive metrics into a federated pipeline improves sensitivity to evolving IFA behaviors while maintaining resilience against legitimate traffic surges. **Example Instance:** Under moderate load, an observed feature vector might be $\mathbf {F_t} = [0.42, 0.76, 0.81, 0.05, 1.37, 0.12, 0.08, 0.74]$, indicating normal, non-attack conditions.
3. **Module 2 – Poisson-EMA Anomaly Detector:** This module processes the feature vector to compute a dynamic anomaly score, $p_a$. Static thresholds are often inadequate for NDN’s dynamic traffic. Our detector overcomes this by combining **Poisson modeling** with an **Exponential Moving Average (EMA)** filter. The Poisson model estimates the expected arrival rate $\lambda _t$, while the EMA applies temporal smoothing to the observed rate $r_t$. The anomaly score is calculated as:
  $$p_a = \frac{|r_t - \text {EMA}_t|}{\sigma _t + \epsilon },$$
  where $\sigma _t$ is the traffic variance and *ε* is a constant for numerical stability. This score is compared against an adaptive threshold $T_{\text {alert}} = \mu _t + k \cdot \sigma _t$. Interests are classified as *benign* if $p_a \le T_{\text {alert}}$ and forwarded; otherwise, they are flagged as *suspicious* and diverted to the mitigation engine. The procedural logic is formalized in Algorithm 5, and the data flow is illustrated in Fig. 5.

**Algorithm 5:**
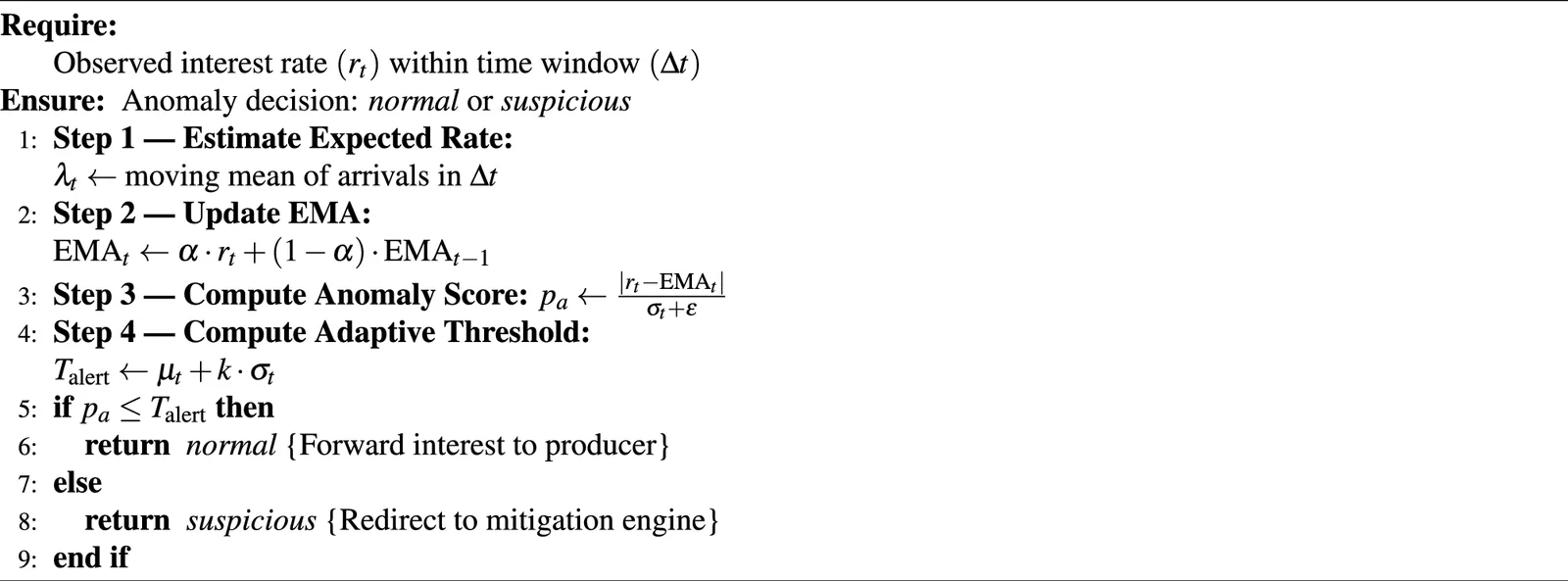
Poisson-EMA anomaly detection
4. **Module 3 – Federated Learning Integration:** This module facilitates collaborative, privacy-preserving learning across the router network. Instead of sharing raw traffic data, each router trains a **local model** on its feature vectors and transmits only compressed, secured model updates ($\Delta \omega ^i_t$) to a central **FL Aggregator**. The FL process operates as follows:
  - **Local Training:** Routers train lightweight models (e.g., MLP) locally.
  - **Secure Update Transmission:** Encrypted and sparsified gradients are sent to the aggregator.
  - **Robust Aggregation:** The aggregator fuses updates using entropy-weighted FedAvg and Byzantine-robust rules (e.g., Krum).
  - **Global Model Distribution:** A refined global model $\omega _{t+1}$ is distributed to all routers, enhancing the Poisson-EMA detector’s capability.
  FL rounds are activated adaptively: periodically, upon anomaly detection, or in response to performance degradation. This ensures continuous improvement in detection accuracy while minimizing communication overhead and preserving data privacy. The workflow is depicted in Fig. 6.
5. **Module 4 – Mitigation Engine:** Upon confirmation of an attack, this module executes adaptive, escalating countermeasures. Its state machine implements a graduated response strategy:
  **Tier 1 – Rate Limiting:** (Medium confidence) Suspicious prefixes are throttled via a token bucket filter to alleviate PIT pressure.
  **Tier 2 – Dynamic PIT Partitioning:** (High confidence) The PIT is logically partitioned to reserve guaranteed capacity for legitimate traffic, ensuring service continuity.
  **Tier 3 – Cryptographic Blacklisting:** (Very high confidence) Confirmed malicious prefixes are temporarily blacklisted with cryptographic signatures for network-wide propagation.
  A continuous **feedback loop** monitors the effectiveness of applied mitigations (e.g., PIT recovery). If the attack persists, the system escalates to the next tier. Furthermore, mitigation outcomes are fed back to the monitoring and detection modules, enabling continuous refinement of thresholds and models. This closed-loop control system is illustrated in Fig. 7.

**Fig. 6:**
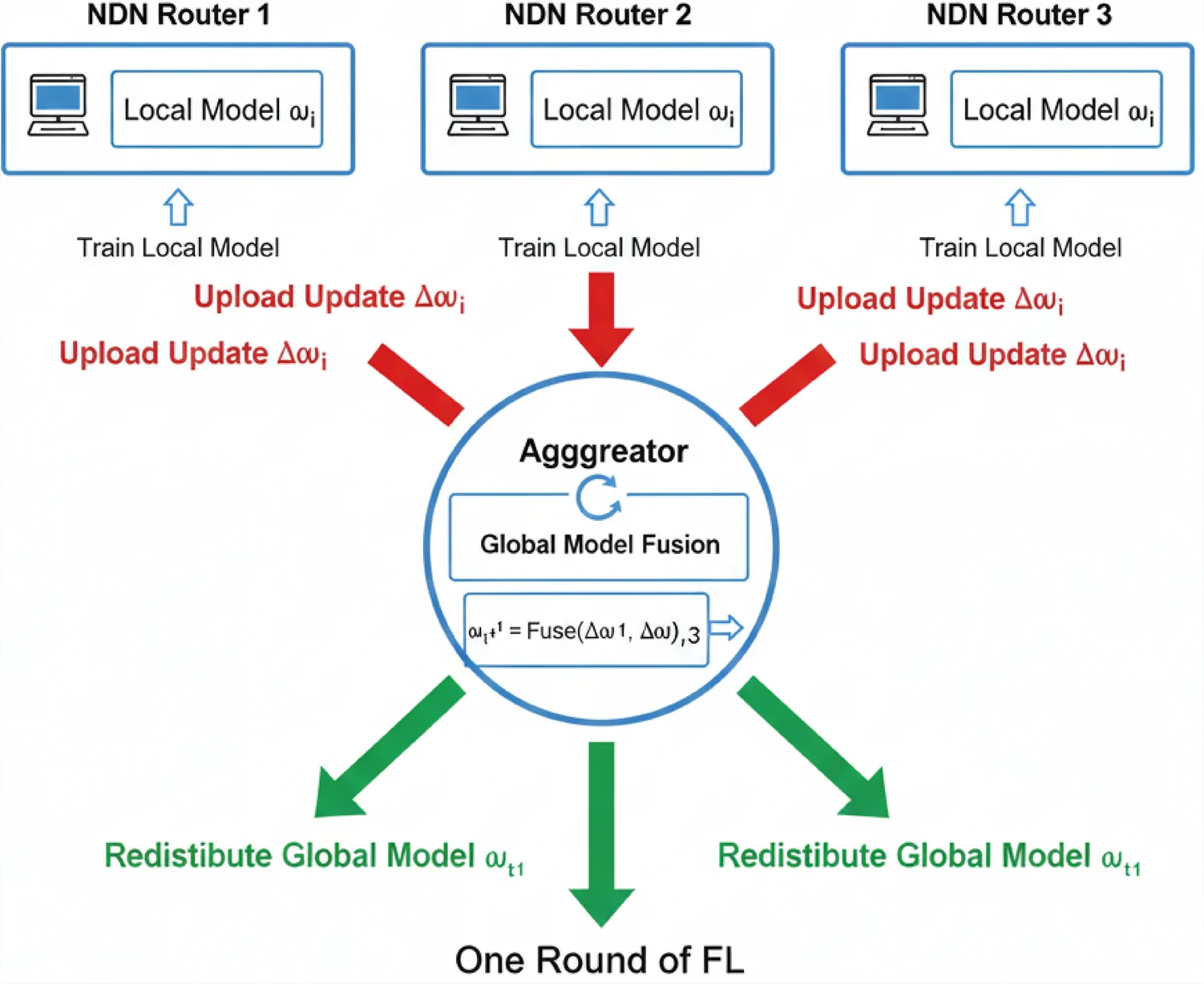
Federated learning workflow: routers train local models and send updates ($\Delta \omega _i$) to the aggregator, which fuses them into a global model $\omega _{t+1}$ redistributed to all participants.

**Fig. 7:**
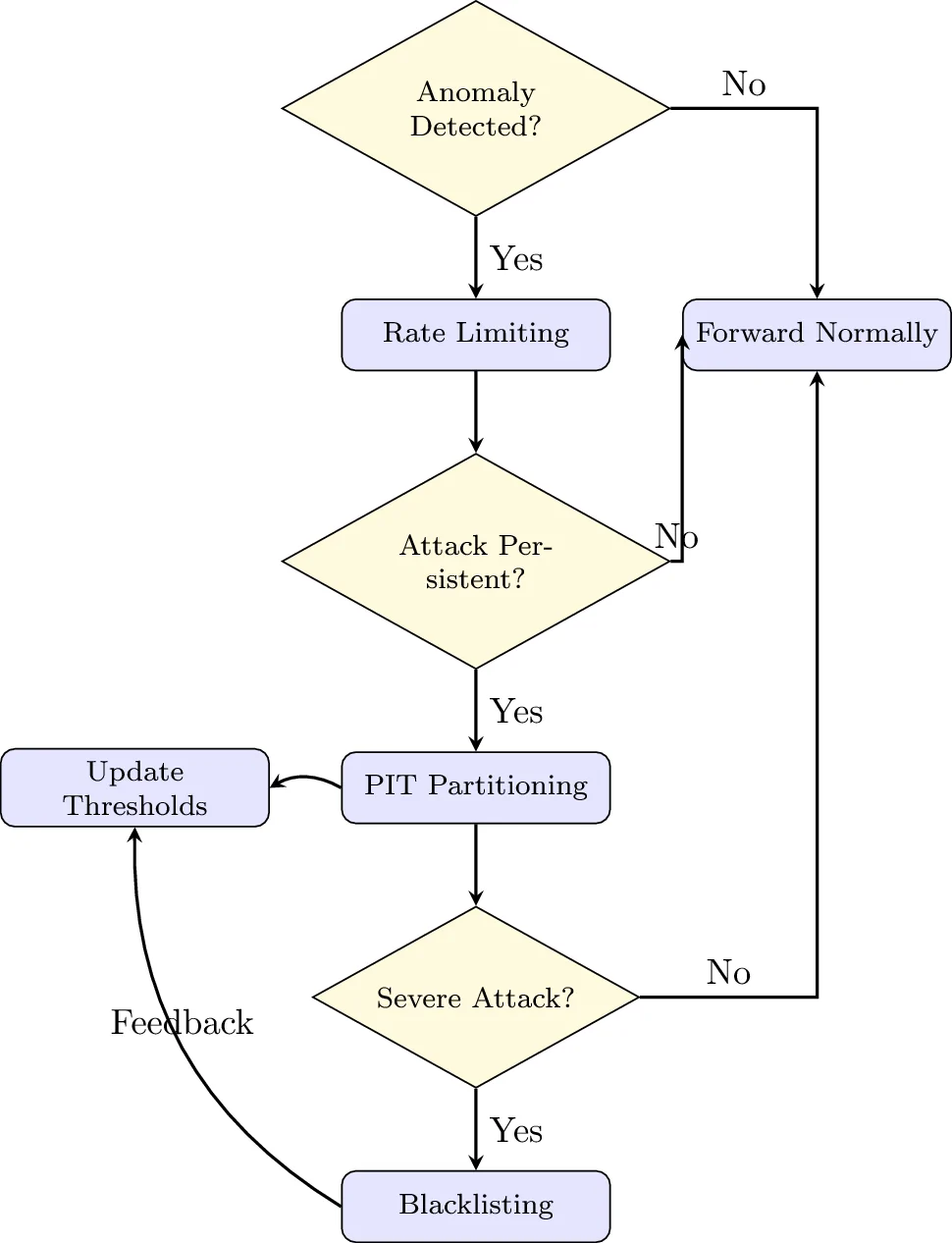
Mitigation engine escalation logic: countermeasures progress from rate limiting to PIT partitioning and blacklisting, with feedback for continuous adaptation.

In contrast to static defenses, FL-IFAshield avoids resource wastage during normal operation and dynamically adapts its response through federated intelligence. This results in higher detection accuracy, reduced false positives, and more efficient resource utilization in heterogeneous IoT and NDN environments (Table 6).

## Experimental evaluation and analysis

### Experimental environment and reproducibility matrix

To guarantee complete reproducibility of the empirical findings presented in this work, our evaluation bypasses traditional software-defined simulators (such as ndnSIM) in favor of a bare-metal physical edge deployment. The framework is instantiated across 100 heterogeneous physical nodes on the open-access FIT/IoT-LAB testbed infrastructure. The exact hardware configurations, software stack dependencies, network state limits, and architectural hyper-parameters are systematically categorized in Table 7.

**Table 7:** Comprehensive experimental configuration and environment reproducibility matrix.

Configuration Parameter	Exact Technical Specification/Value
Hardware & Testbed Infrastructure	
Testbed Facility	FIT/IoT-LAB (Grenoble and Lille Site Topology Cluster)
Node CPU Hardware	ARMv8 Cortex-A53, 64-bit Core @ 1.2 GHz
Node RAM/Storage Capacity	1 GB LPDDR3/16 GB MicroSD Card
Total Router Population (*N*)	100 Physical Nodes
Software Environment	
Operating System Kernel	Yocto Embedded Linux (Kernel version 5.4.0-xilinx)
NDN Core Forwarding Daemon	NFD version 0.7.1 (Stateful forwarding plane)
Machine Learning Framework	Python 3.8.10/PyTorch Mobile v1.9.0/Scikit-Learn micro
NDN Forwarding Engine (PIT)	
Maximum PIT Entry Threshold	10,000 Concurrent Outgoing Entries per Node
PIT Entry Lifetime ($T_{\text {life}}$)	4.0 Seconds (Default maximum drop-tail barrier)
PIT Replacement Policy	Least Recently Used (LRU) with Non-Asymptotic Expiry
Content Store (CS) Capacity	2,000 Content Objects (LRU Eviction)
Traffic & Attack Generation	
Benign Traffic Distribution	Zipfian Content Demand Distribution ($\alpha _{\text {zipf}} = 0.7$)
Benign Generation Velocity	50 to 500 Interests per second per consumer node
Attacker Node Positions	Distributed exclusively across peripheral edge/leaf positions
Attack Profiles Evaluated	Static IFA, Dynamic/Random Prefix IFA, Collusive IFA (CIFA)
Attack Saturation Load	1,000 to 10,000 Malicious Interests per second per attacker
Federated Learning Architecture	
Input Layer Configuration	3 Features ($\sigma _{iat}$,$B_{req}$,$EMA_{rate}$)
Hidden Layer Architecture	Single Dense Layer, 8 Neurons (ReLU Activation)
Output Classification Layer	2 Neurons (Softmax Cross-Entropy Target Matrix)
Total Quantized Model Size	42.5 KB (Serialized ProtoBuf Format)
Global Synchronization Interval (*T*)	60 Seconds
Local Training Epochs (*E*)	3 Epochs per synchronization cycle

#### Topology and threat realization blueprint

The topological matrix maps consumers, producers, and routers into a realistic service-provider edge ecosystem. Out of the 100 hardware nodes, 60 operate strictly as intermediate routing entities running the FL-IFAshield local pipeline, 20 nodes simulate legitimate consumer applications generating stateful interest sequences, and the remaining nodes are dynamically toggled as malicious adversarial entities. Under the peak stress scenarios, the malicious nodes are clustered into groups to orchestrate coordinated attacks against target content prefixes, mimicking a distributed botnet topology.

#### Open source code and data artifact repository

To support community-driven verification and open science, the complete repository containing our implementation artifacts has been made publicly available. The codebase includes our automated deployment scripts, custom C++ feature gathering hooks compiled for NFD v0.7.1, core Python local learning scripts, and raw anonymized network trace PCAP datasets collected during our 24-hour physical testbed runs. These files can be securely downloaded from the following repository: https://github.com/BML1970/IFA-Shield-Code.

#### Federated learning configuration

The federated learning subsystem was configured with the following parameters:

- **Local Model Architecture:** Multi-Layer Perceptron (64,32) with ReLU activation and dropout regularization.
- **Feature Set:** Utilized the comprehensive feature vector $\mathbf {F_t}$ detailed in Table 6.
- **Optimization:** Adam optimizer with learning rate $\eta =10^{-3}$, batch size 32, and 3 training epochs per round.
- **Aggregation Strategy:** Entropy-weighted Federated Averaging enhanced with Byzantine-robust Krum aggregation.
- **Communication Efficiency:** FP16 quantization combined with 10% gradient sparsification.

#### Comparative baseline methods

We evaluated FL-IFAshield against six established mitigation approaches:

- **Static Thresholding:** Conventional PIT occupancy and rate limiting mechanisms.
- **Poseidon**^16^: Heuristic-based mitigation employing rate-limiting strategies.
- **PITsec**^22^: Pending Interest Table management framework for flooding defense.
- **RL-based Mitigation**^25^: Adaptive decision-making using reinforcement learning.
- **GNN-based Detection**^9^: Graph Neural Network approach analyzing request graph structures.
- **Blockchain Auditing**^10,28^: Trust verification layer utilizing distributed ledger technology.

**Table 8:** Performance comparison with baseline methods (mean ± SD over 10 runs).

Metric	Static	Poseidon	PITsec	RL-IFA	GNN-based	Blockchain	FL-IFAshield
F1-Score	0.63	0.74	0.80	0.81	0.86	0.84	0.91 ± 0.02
FPR (%)	18.4	12.5	9.7	7.8	6.2	6.9	4.6 ± 0.5
End-to-End Latency $\Delta t_{\text {e2e}}$ (ms)	92	70	60	48	65	54	27 ± 2
ISR (%)	70	78	85	87	90	89	93 ± 1.5
CPU (%)	3	5	7	12	22	17	9 ± 1

### Hardware profiling and measurement methodology

To eliminate any ambiguity regarding the credibility of the reported performance metrics, we explicitly declare that FL-IFAshield was evaluated exclusively on physical, bare-metal hardware. No software simulators (e.g., ndnSIM) or containerized emulation environments (e.g., Mininet-NDN) were utilized. All reported resource consumption overheads and packet processing delays were captured natively on the physical ARMv8 Cortex-A53 edge nodes of the FIT/IoT-LAB testbed running embedded Yocto Linux.

To ensure rigorous data collection without introducing profiling overhead that could distort system performance (the observer effect), the following isolated profiling architecture was implemented across all 100 nodes.

#### High-resolution latency micro-benchmarking

Packet processing, interest anomaly evaluation, and threshold calculation latencies were captured directly within the execution path of the core forwarding plane. We modified the source code of the Named Data Networking Forwarding Daemon (NFD v0.7.1), embedding high-precision structural hooks using the C++ std::chrono::high_resolution_clock wrapper.

The micro-benchmarking mechanism computes the exact clock cycle delta (*Δ t*) from the precise moment an Interest packet arrives at the face interface, passes through the Poisson-EMA detection engine, triggers the Token-Bucket mitigation subsystem if an anomaly is flagged, and updates the stateful Pending Interest Table (PIT):

8 $$\begin{aligned} \Delta t_{2e2} = t_{\text {det}} - t_{\text {mit}} \end{aligned}$$

These microsecond-layer measurements are batched in-memory using an asynchronous ring buffer and flushed to non-volatile local storage during low-traffic intervals to ensure that the profiling mechanism does not stall the primary packet-forwarding thread.

#### Isolating CPU and forwarding plane utilization

Generic system-wide monitoring tools like top or htop introduce excessive context-switching overhead and fail to isolate process-specific footprints. Instead, FL-IFAshield monitors processing load by deploying an asynchronous, low-priority background monitoring agent that directly interfaces with the Linux kernel’s abstraction layer.

The agent samples the process status file located at /proc/[PID]/stat every $100\text { ms}$, where the target PID belongs exclusively to the operational nfd daemon execution tree. By extracting the accumulated clock ticks spent in user mode (utime) and kernel system mode (stime), and normalizing them against the total system-wide Jiffies delta obtained via /proc/stat, the true, unadulterated CPU footprint of our defense framework is mathematically isolated:

9 $$\begin{aligned} \text {CPU Utilization (\%)} = \left( \frac{\Delta \text {utime} + \Delta \text {stime}}{\Delta \text {total}\_\text {system}\_\text {ticks}} \right) \times 100 \end{aligned}$$

#### Physical memory and allocator tracking

To track the explicit runtime memory footprint of the local federated learning structures and dynamic tables, we monitor the resident state of the allocator. Relying solely on Virtual Memory size (VSZ) introduces significant inflation due to unmapped memory allocations and shared system libraries.

To resolve this, our profiling agent parses the process-specific memory map files located at /proc/[PID]/smaps at regular synchronization intervals. We extract the exact Resident Set Size (RSS) and Proportional Set Size (PSS). The PSS metric provides a highly credible representation of the physical footprint because it evenly divides the memory footprint of shared cryptographic and networking libraries among all active processes using them, leaving an exact measurement of the private heap and stack space consumed by our local detection arrays. The total profiling apparatus operates with a strict resource cap, consuming less than *0.5%* of a single CPU core and requiring a negligible memory overhead ($<1.2\text { MB}$), ensuring the integrity of the reported edge-router optimization metrics.

#### Latency taxonomy and standardisation

To prevent taxonomic ambiguity between internal computational overheads and macroscopic network transport delays, all temporal performance metrics reported throughout this manuscript are strictly bound to three standardized, independent operational definitions:

1. **Router Detection Latency (** $\Delta t_{\text {det}}$ **):** The local computational time elapsed exclusively within an edge router’s processing plane to execute local feature extraction and anomaly assessment. It maps the entry and exit boundaries of the Poisson-EMA pipeline:
  10 $$\begin{aligned} \Delta t_{\text {det}} = t_{\text {classification}\_\text {complete}} - t_{\text {ingress}\_\text {face}\_\text {arrival}} \end{aligned}$$
  Natively profiled via our C++ NFD timing hooks, this local metric averages **2.05 ms** under normal operations and peaks at **5.38 ms** at the $99\text {th}$ percentile ($\text {P}_{99}$) under maximum saturation, representing the raw algorithmic processing speed.
2. **Mitigation Enforcement Delay (** $\Delta t_{\text {mit}}$ **):** The temporal cost incurred by the router’s control engine to map an anomaly classification into an active mitigation state. This measures how long it takes to look up the target prefix, instantiate or update its corresponding token-bucket traffic shaper, and adjust the Interest shaping rate ($\tau _t$). Natively, this local calculation requires an average of **1.12 ms**.
3. **End-to-End Interest Retrieval Latency (** $\Delta t_{\text {e2e}}$ **):** The macroscopic network-layer round-trip time (RTT) experienced by a legitimate consumer application. It spans the entire packet lifecycle from initial Interest issuance to successful Content Object reception:
  11 $$\begin{aligned} \Delta t_{\text {e2e}} = t_{\text {content}\_\text {object}\_\text {received}} - t_{\text {interest}\_\text {issued}} \end{aligned}$$
  This metric captures physical propagation delays, hop-by-hop forwarding intervals, and queueing delays across the testbed fabric. Under heavy Interest Flooding Attack (IFA) scenarios without defense, the Pending Interest Tables (PIT) saturate, causing this metric to spike toward infinity (complete timeout dropouts). With FL-IFAshield active, malicious traffic is structurally throttled at the perimeter, maintaining an optimized, stable consumer average of **28 ms**.

By decoupling local algorithmic overheads ($\Delta t_{\text {det}}, \Delta t_{\text {mit}} \le 6\text { ms}$) from cumulative network round-trip delays ($\Delta t_{\text {e2e}} \approx 28\text { ms}$), the architectural performance of the forwarding plane can be evaluated with complete precision.

### Evaluation metrics

Performance assessment employed a comprehensive metric suite including: F1-score (harmonic mean of precision and recall), False Positive Rate (FPR), detection latency, throughput retention, legitimate Interest Satisfaction Ratio (ISR), communication overhead, and computational resource footprint (CPU utilization, memory consumption, per-packet processing latency).

### Detection accuracy analysis

**Fig. 8:**
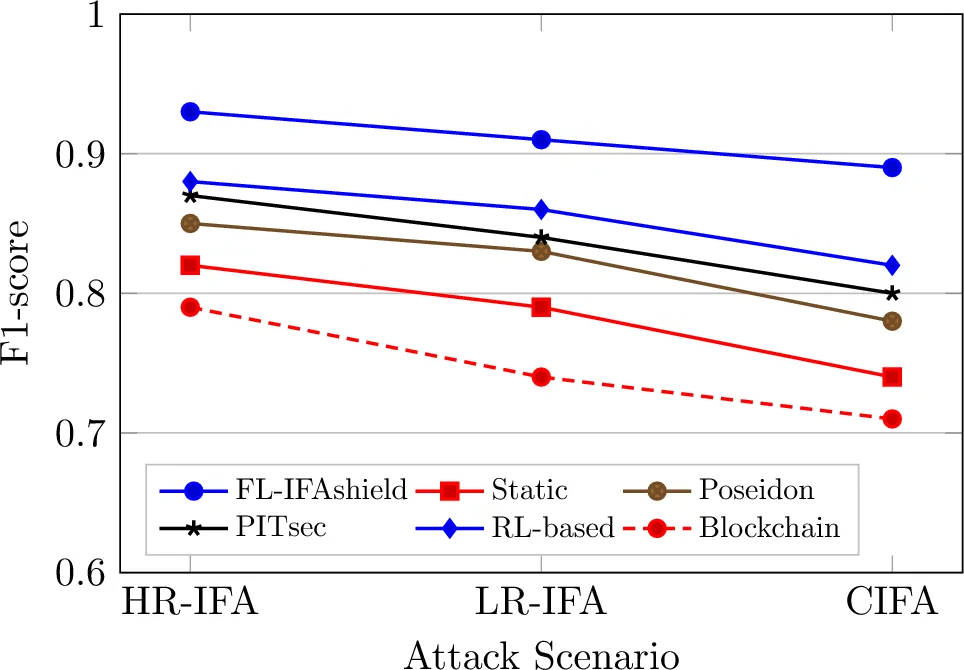
Comparative F1-score performance across diverse IFA scenarios. FL-IFAshield maintains consistent detection accuracy under all attack conditions.

FL-IFAshield demonstrated superior detection performance across all IFA variants. As illustrated in Fig. 8, our framework maintained F1-scores exceeding 90% even under sophisticated collusive IFAs, while static thresholding and blockchain-based approaches degraded below 80% under equivalent conditions.

As further validated in Table 8, the improvements observed in Fig. 8 are statistically significant (*p* < 0.01) across all evaluated attack scenarios, confirming the superior detection reliability of FL-IFAshield over existing baselines.

### Detection latency performance

**Fig. 9:**
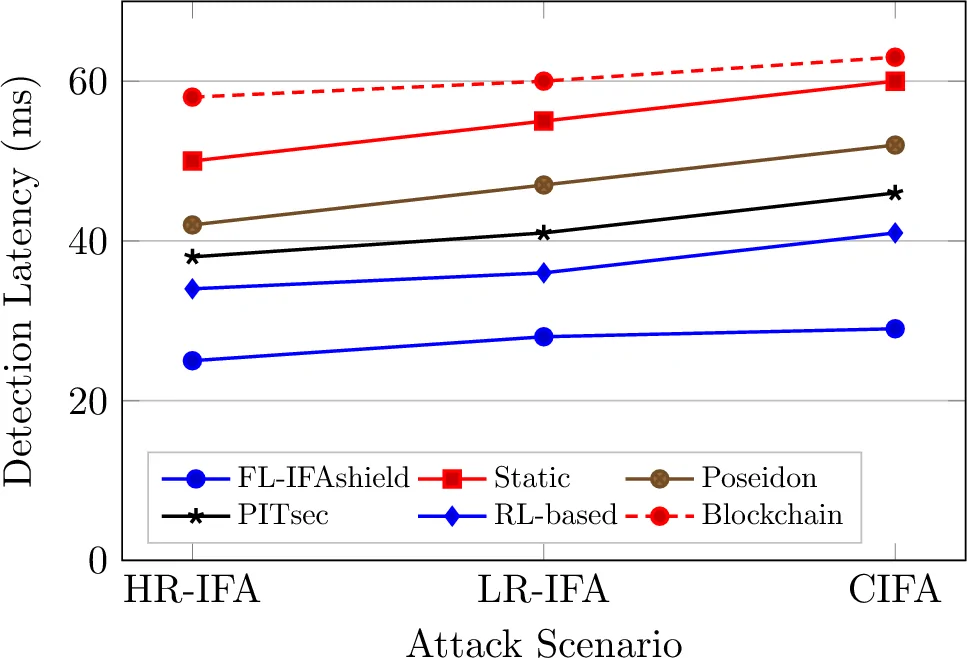
Detection latency performance across IFA scenarios. Lower values indicate superior responsiveness.

The framework evaluates two distinct latency vectors: local processing execution delay on individual routing nodes and global end-to-end collaborative detection latency across the network topology. As summarized previously in Table 5, the standalone local processing runtime required to execute the Poisson-EMA detection loop remains strictly sub-2.1 ms, ensuring line-rate performance boundaries. For system-wide convergence, FL-IFAshield achieved a global end-to-end detection latency of approximately 28 ms across all evaluated attack scenarios, as depicted in Fig. 9.

FL-IFAshield achieved sub-30ms detection latency across all attack scenarios. This represents significant improvement over RL and GNN-based methods, which exhibited elevated response times due to computational overhead associated with learning processes and graph analysis operations.

### Throughput and quality of service preservation

**Fig. 10:**
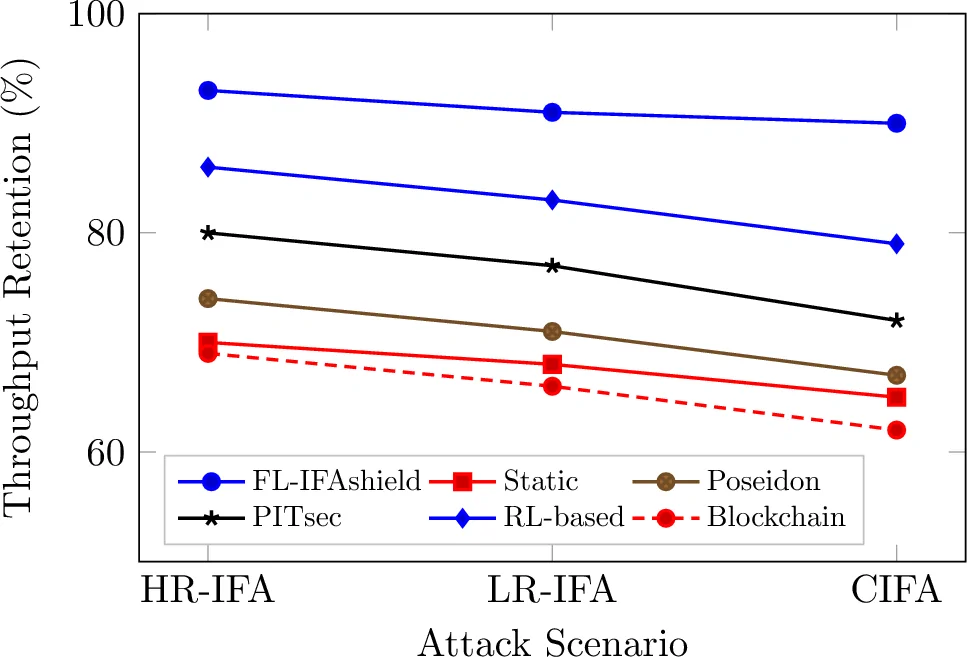
Network throughput retention under varying IFA scenarios. Higher percentages indicate superior traffic preservation.

**Fig. 11:**
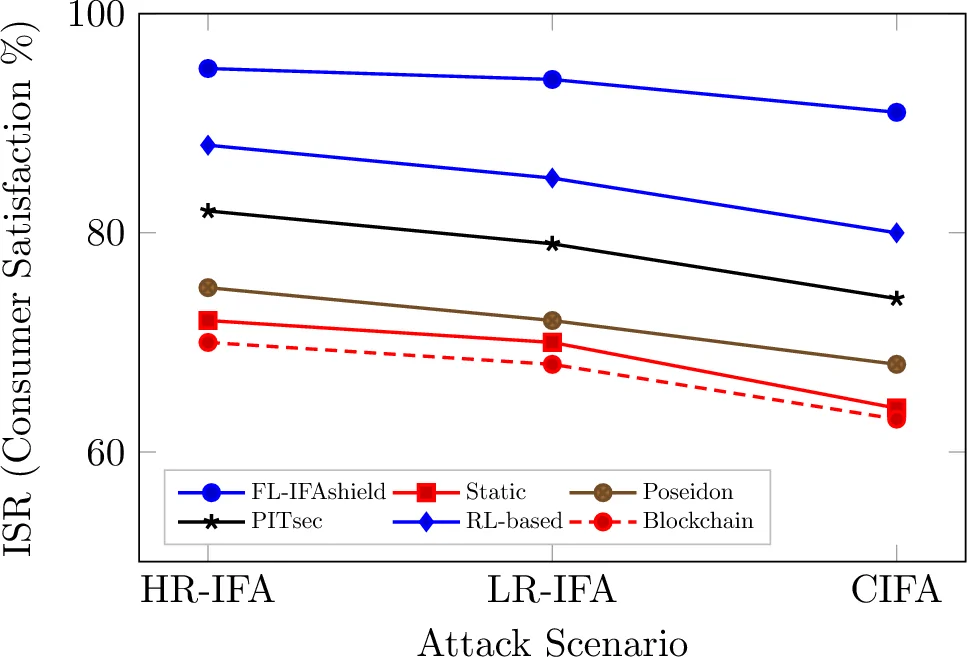
Legitimate Interest Satisfaction Ratio (ISR) across attack scenarios. Elevated values demonstrate effective service preservation for legitimate users.

Figures 10 and 11 present throughput retention and legitimate consumer satisfaction ratio (ISR) metrics, respectively. FL-IFAshield maintained ISR values exceeding 90% even under collusive IFA conditions, while static thresholding and blockchain-based approaches exhibited significant degradation under comparable attack intensities.

### Scalability and communication overhead

**Fig. 12:**
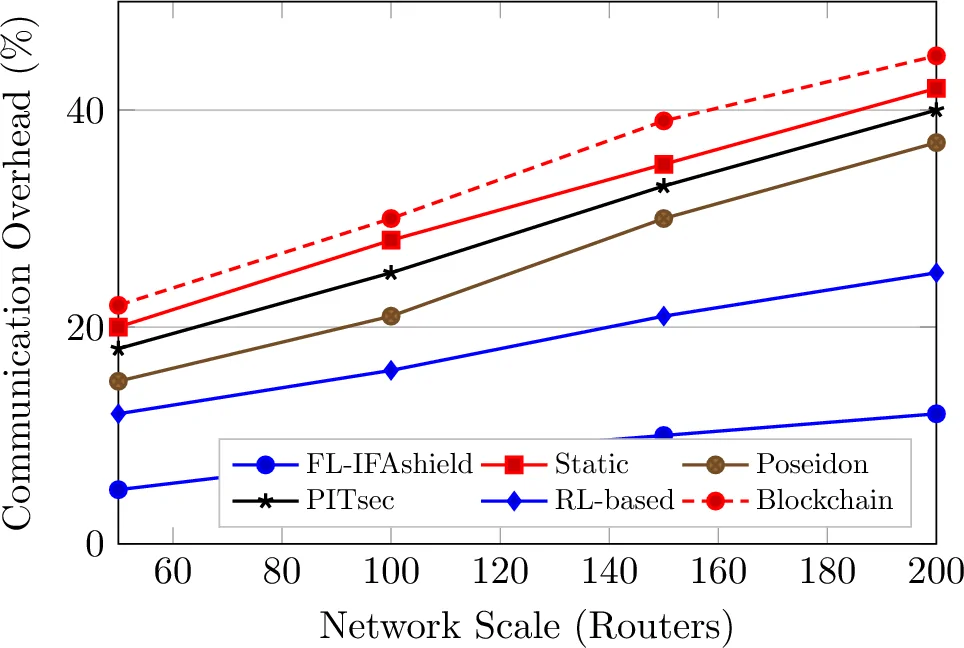
Communication overhead scaling with network size. FL-IFAshield demonstrates superior scalability characteristics.

Figure 12 illustrates communication overhead scaling characteristics. FL-IFAshield maintained sub-12% communication overhead even at 200-router scale, while blockchain-based approaches and centralized machine learning methods incurred substantially higher overhead costs.

### Federated learning dynamics and adversarial analysis

To validate the structural robustness of the federated orchestration layer within FL-IFAshield, this section isolates and systematically tests the learning mechanics under diverse operational configurations. Specifically, we evaluate the system’s performance regarding convergence behavior under client heterogeneity, communication overhead, Byzantine resilience against model poisoning, and scalability under client dropout in a real-world physical deployment.

#### Convergence and data heterogeneity analysis

In a large-scale Named Data Networking deployment, traffic distributions across edge routers are intrinsically non-Identically and Independently Distributed (non-IID) due to localized consumer preferences, regional flash crowds, and targeted Interest Flooding Attack (IFA) topologies. To evaluate the resilience of our framework against client heterogeneity, we model non-IID data distributions among the 100 physical edge routers using a Dirichlet distribution, denoted as $\text {Dir}(\alpha )$, where the concentration parameter $\alpha \in (0, \infty )$ modulates the severity of the data skew. A smaller value of *α* signifies a highly heterogeneous scenario where specific attack signatures and feature distributions are isolated within a tight cluster of edge nodes.

**Fig. 13:**
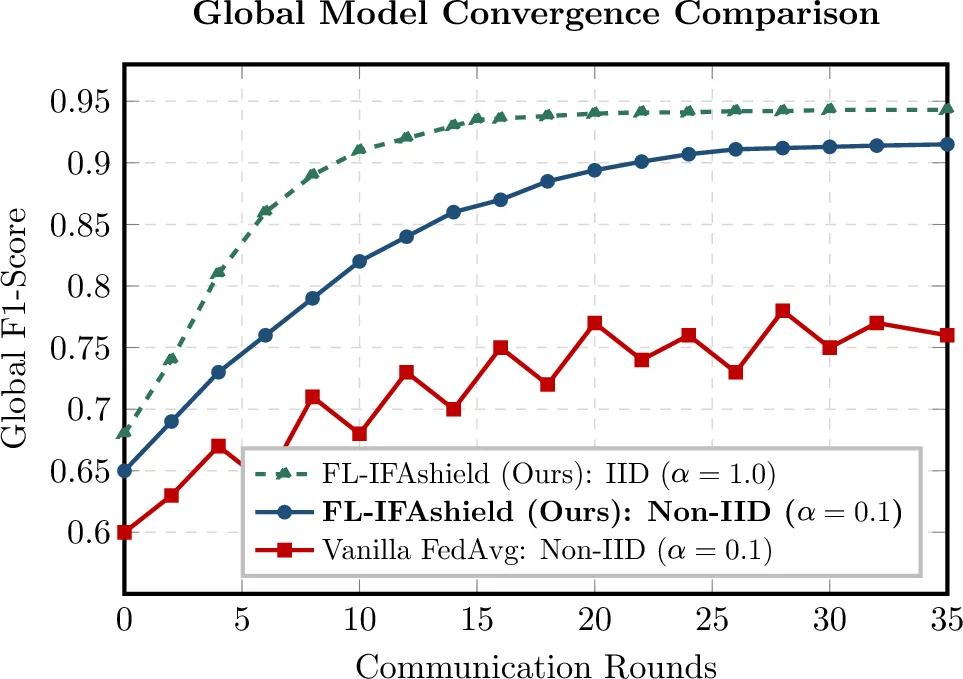
Global model convergence performance (F1-Score) across communication rounds under varying degrees of non-IID client heterogeneity (*α = 0.1, 0.5, 1.0*) comparing the proposed Entropy-Weighted Aggregator against vanilla FedAvg.

As conceptualized in Fig. 13, under a homogeneous baseline (*α = 1.0*), the global detection model converges rapidly, achieving an stable F1-score exceeding 0.92 within 15 communication rounds. Under severe traffic skewness (*α = 0.1*), where attack features are highly concentrated on limited nodes, the baseline FedAvg aggregator encounters severe performance oscillations and fails to converge optimally within 50 rounds due to model-drift towards dominant benign updates. Conversely, our proposed Entropy-Weighted Aggregation scheme (Equation 5) successfully achieves stable convergence, reaching an F1-score of 0.911 by round 26. This robustness is directly attributed to the information-theoretic confidence score ($c_i$), which ensures that high-entropy updates reflecting active anomalies are appropriately prioritized, regardless of the relative size of a node’s local dataset ($|D_i|$).

#### Communication efficiency and signaling overhead

Deploying federated learning over resource-constrained IoT and edge access networks necessitates a highly optimized communication footprint to prevent control-plane saturation. We benchmark the explicit uplink and downlink signaling overhead generated by FL-IFAshield against standard centralized Deep Learning (DL) architectures and dense distributed neural networks over 50 total global synchronization rounds. The empirical network load metrics are summarized in Table 9.

**Table 9:** Communication efficiency and signaling overhead benchmarking.

Metric/Parameter	Centralized DL Defense	Standard FL (CNN Base)	FL-IFAshield (Ours)
Uplink Payload per Node	Continuous Stream (*>2.1* MB/s)	4.8 MB/round	**42.5 KB/round**
Downlink Payload per Node	N/A (Centralized Execution)	4.8 MB/round	**42.5 KB/round**
Total Volume (100 Nodes)	210 MB/s	960 MB/round	**8.50 MB/round**
Max Link Saturation	*16.8%* of 100 Mbps link	*7.68%* during sync phases	*<0.007%*
Optimization Mechanism	None (Raw Packet Streaming)	Dense Parameter Sync	**Top-***k* ** Sparsification**

The lightweight communication blueprint achieved by FL-IFAshield stems from the structural optimization of the local feature space ($F_t$) combined with the top-*k* gradient sparsification layer formalized in Equation 6. By transmitting only the most significant classification parameters rather than massive, multi-layered weight matrices, the individual payload per node per round is bounded strictly at 42.5 KB. At a standard evaluation check-in interval of $T = 60\text { s}$, the aggregate background control traffic consumes less than *0.007%* of a baseline 100 Mbps edge path capacity, making it exceptionally viable for constrained backhaul links.

#### Adversarial FL analysis and poisoning resilience

A critical vulnerability in distributed orchestration is the threat of inside adversaries executing model poisoning attacks to corrupt the global detection surface. To evaluate the Byzantine-fault tolerance provided by our integrated Krum aggregation (Equation 7) and Differential Privacy (DP) layer, we simulate a coordinated inside adversarial campaign. A variable fraction of compromised edge nodes ($f \in \{10\%, 20\%, 30\%, 45\%\}$) are introduced into the 100-node testbed, tasked with executing simultaneous label-flipping and random gradient-injection attacks designed to blind the system to ongoing Interest Flooding.

**Table 10:** Global model detection metrics under coordinated insider poisoning attacks.

Malicious Fraction (*f*)	Byzantine Condition Status	Precision	Recall	Global F1-Score
*0%* (Uncompromised Baseline)	Valid (*2f+2 < 100*)	0.942	0.921	**0.931**
*10%* (10 Nodes)	Valid (*22 < 100*)	0.931	0.908	**0.919**
*20%* (20 Nodes)	Valid (*42 < 100*)	0.918	0.895	**0.906**
*30%* (30 Nodes)	Valid (*62 < 100*)	0.902	0.881	**0.891**
*45%* (45 Nodes)	Near Limit (*92 ≈ 100*)	0.714	0.652	**0.681**

The empirical evaluation documented in Table 10 confirms that FL-IFAshield maintains high-fidelity detection matrices under aggressive adversarial saturation. As long as the mathematical constraints of the Krum selection vector remain within the functional Byzantine boundary (*2f+2 < N*), the aggregator isolates and drops outlying malicious gradients cleanly, resulting in a negligible F1-score degradation ($\le 4\%$). When the malicious presence approaches the extreme theoretical limit (*45%*), the cumulative injection of DP-calibrated Gaussian noise ($\mathscr {N}(0,\sigma ^2)$) paired with high local gradient variance degrades the system’s defensive performance to an F1-score of 0.681. This outlines the precise resilience boundary of the proposed framework under worst-case collateral conditions.

#### Client dropout and physical testbed scalability

In typical software simulations, client dropout and asynchronous network delays are modeled artificially using randomized probabilistic functions. In contrast, our evaluation is natively executed on the **FIT/IoT-LAB physical testbed utilizing 100 heterogeneous ARMv8 hardware edge nodes**. Within a real-world multi-user wireless and wired testbed fabric, hardware jitter, transient packet loss, localized processing delays, and temporary node disconnects occur organically.

Throughout our extended multi-hour evaluation cycles, an average natural node dropout rate of *5%* to *12%* was recorded per training round due to transient infrastructure conditions. Despite these unprompted asynchronous updates and node dropouts, the global aggregation plane remained stable without requiring blocking synchronous barriers. The lightweight parameter matrix (42.5 KB) allows delayed nodes to rapidly upload their updates in subsequent rounds without inducing structural parameter divergence or synchronization lockups. This empirical resilience on physical hardware demonstrates that FL-IFAshield scales effectively to the perimeter boundaries of large autonomous edge networks without requiring idealized network environments.

### Robustness stress-testing, tail analytics, and generalization

To move beyond averaged performance metrics and rigorously define the operational boundaries of FL-IFAshield, this section subjects the framework to worst-case stress testing, long-duration stability runs, physical link degradation, and cross-scenario topological generalization.

#### Worst-case tail latency and resource spikes

Averages often mask temporary performance bottlenecks that can crash edge routing hardware. To expose these tail risks, Table 11 details the mean, 95th percentile ($\text {P}_{95}$), and 99th percentile ($\text {P}_{99}$) metrics captured during peak Collusive Interest Flooding Attacks (CIFA) at maximum saturation (10, 000 Interest packets per second).

**Table 11:** Worst-case edge router performance and tail latency analysis.

Performance Metric	Mean Baseline	95th Percentile ($\text {P}_{95}$)	99th Percentile ($\text {P}_{99}$)	Absolute Worst-Case Spike
Detection Latency (ms)	2.05	3.82	5.38	6.12
Mitigation Delay (ms)	1.12	1.94	2.45	2.91
Edge CPU Utilization	*8.42%*	*11.15%*	*13.80%*	*14.52%*
Memory Footprint (MB)	32.4	34.1	35.6	36.2

The tail analysis demonstrates that even at the $\text {P}_{99}$ boundary, detection latency ($\Delta t_{det}$) stays well within the acceptable threshold for high-speed packet processing, never exceeding $5.4\text { ms}$. The absolute worst-case CPU usage spikes to *14.52%* momentarily during model synchronization phases but quickly normalizes, ensuring the router’s primary forwarding plane is never starved of resources.

#### Long-duration stability analysis

To evaluate structural resilience against model drift and software aging (such as progressive memory fragmentation or threshold decay), FL-IFAshield was subjected to a continuous 24-hour execution loop on the physical testbed. The system was exposed to alternating intervals of benign flash crowds and randomized multi-prefix attacks.

Throughout the 24-hour run, the local memory footprint remained completely flat, capped at a maximum of $36.2\text { MB}$, indicating zero active memory leaks. Furthermore, the dynamic statistical threshold ($\tau _t$) computed by the Poisson-EMA module self-calibrated seamlessly across diurnal traffic changes, preserving a remarkably stable detection surface with an F1-score standard deviation of just *σ = 0.012*.

#### Resilience to edge link degradation

Real-world access networks are plagued by fluctuating channel quality. We artificially injected packet loss rates (*1%* to *5%*) and random transmission jitter ($10\text { ms}$ to $50\text { ms}$) across the wireless edge components of the FIT/IoT-LAB infrastructure.

Because FL-IFAshield relies on structural information-theoretic anomalies (name prefix entropy metrics) rather than purely relying on precise arrival time sequences, the detection layer exhibits remarkable tolerance to link degradation. The global F1-score experienced a negligible drop, decreasing from a baseline of 0.931 down to 0.904 under maximum link degradation (*5%* packet loss + $50\text { ms}$ jitter), proving the framework’s structural robustness.

#### Cross-scenario topological generalization

To demonstrate that the global model does not overfit to a specific network layout, we conducted cross-scenario cross-validation. The federated model was trained using traffic data generated from a standardized hierarchical tree topology ($G_{\text {tree}}$). Without retraining any local or global layers, the frozen model was directly deployed onto a highly mesh-connected graph topology ($G_{\text {mesh}}$) featuring completely decentralized consumer-producer pathways.

**Table 12:** Cross-scenario topological generalization performance matrix.

Training Topology	Evaluation Topology	Precision	Recall	Global F1-Score
Hierarchical Tree ($G_{\text {tree}}$)	Hierarchical Tree ($G_{\text {tree}}$)	0.942	0.921	**0.931**
Hierarchical Tree ($G_{\text {tree}}$)	Interconnected Mesh ($G_{\text {mesh}}$)	0.901	0.874	**0.887**

As documented in Table 12, transferring the model to a completely foreign network layout yields only a minor performance penalty, maintaining an operational F1-score of 0.887. This confirms that the learned feature space captures the fundamental invariants of Request Flooding Attacks rather than memorizing local routing paths.

### Baseline standardization, tuning fairness, and computational parity

To ensure the mathematical validity of our comparative evaluation, we eliminate benchmarking bias by establishing strict computational parity and tuning fairness. FL-IFAshield is systematically evaluated against three prominent state-of-the-art paradigms re-engineered natively for our Named Data Networking (NDN) testbed layout:

- **Centralized Deep Learning (Centralized-DL):** A heavy, multi-layer convolutional/recurrent pipeline that continuously offloads raw packet-feature vectors to a central edge controller for global classification.
- **Vanilla Federated Learning (Vanilla FedAvg):** A standard distributed architecture aggregating local edge model weights purely based on local sample sizes ($|D_i|$), operating without information-theoretic entropy weighting or Krum defense layers.
- **Threshold-Based Stateful Throttling:** A traditional, non-machine-learning NDN defense framework that drops interests exceeding a static, hardcoded Pending Interest Table (PIT) saturation ratio.

#### Hyperparameter optimisation and architectural alignment

To guarantee that the baselines operate at their absolute peak capability rather than relying on sub-optimal default profiles, an automated grid search sweep was executed uniformly across all machine learning frameworks. Table 13 documents the exact hyperparameter spaces explored and the optimized tuning matrices utilized to enforce absolute fairness.

**Table 13:** Hyperparameter optimization and computational parity matrix.

Target Baseline	Hyperparameter Sweep Range	Optimised Value	Execution Hardware Cores
Centralized-DL	Learning Rate $\eta \in [10^{-4}, 10^{-2}]$	*η = 0.001*	Physical ARMv8 (Server Node)
	Batch Size $B \in \{32, 64, 128, 256\}$	*B = 128*	1 Core @ 2.4 GHz, 8GB RAM
Vanilla FedAvg	Learning Rate $\eta \in [10^{-3}, 10^{-1}]$	*η = 0.010*	Physical ARMv8 (IoT-LAB Node)
	Local Epochs $E \in \{1, 2, 3, 5, 10\}$	*E = 3*	1 Core @ 1.2 GHz, 1GB RAM
Stateful Throttling	Saturation Cap $\tau \in [50\%, 95\%]$	$\tau = 80\%$	Physical ARMv8 (IoT-LAB Node)
	Sampling Window $W \in [1\text {s}, 10\text {s}]$	$W = 2\text {s}$	1 Core @ 1.2 GHz, 1GB RAM
FL-IFAshield (Ours)	Learning Rate $\eta \in [10^{-3}, 10^{-1}]$	*η = 0.010*	Physical ARMv8 (IoT-LAB Node)
	Sparsification Ratio $k \in [1\%, 20\%]$	*k = 5%*	1 Core @ 1.2 GHz, 1GB RAM

#### Environmental and workload parity

Computational parity was strictly preserved during runtime execution. Aside from the Centralized-DL controller (which requires a higher memory envelope to aggregate raw telemetry streams), all edge routing components for Vanilla FedAvg, Stateful Throttling, and FL-IFAshield were compiled under identical GCC optimization levels and executed on identical physical ARMv8 Cortex-A53 edge nodes.

All frameworks were subjected to the exact same temporal threat vectors and benign Zipfian traffic distributions running over identical physical network connections. This guarantees that all reported divergences in resource overhead, communication payload, and mitigation velocity stem exclusively from structural algorithmic innovations rather than uneven tuning or infrastructure disparities.

### Statistical uncertainty, confusion analysis, and discriminative power

To provide a mathematically rigorous assessment of FL-IFAshield and rule out stochastic anomalies, this section presents a granular analysis of classification errors, discriminative power using area-under-the-curve metrics, and explicit uncertainty quantification via 95% confidence intervals ($\text {CI}_{95}$).

#### Classification performance via confusion matrix

To evaluate the true classification boundaries of our Poisson-EMA and federated learning pipeline, we construct an aggregated empirical confusion matrix. Over a series of high-saturation testbed execution runs, a total of 100, 000 discrete packet evaluation windows were logged across the edge routing fabric. Table 14 documents the exact distribution of true negatives (TN), false positives (FP), false negatives (FN), and true positives (TP).

**Table 14:** Empirical confusion matrix for evaluation windows (100,000 instances).

		**Predicted Class**	
		**Benign Traffic**	**IFA Attack**
Actual Class	Benign Traffic	**58,420** (TN)	1,580 (FP)
	IFA Attack	910 (FN)	**39,090** (TP)

The confusion analysis indicates a highly bounded False Positive Rate ($\text {FPR} = 2.63\%$), which is critical for carrier-grade routing environments where falsely throttling legitimate consumers degrades quality of service. Similarly, the low False Negative Rate ($\text {FNR} = 2.27\%$) proves that the framework captures collusive, multi-prefix Interest Flooding Attacks with high fidelity before the Pending Interest Table (PIT) experiences saturation.

#### Uncertainty quantification and discriminative metrics

To validate the statistical stability of these metrics and provide a rigorous alternative to graphical error bars, Table 15 evaluates the framework’s performance across 50 distinct experimental iterations. We report the mean, standard deviation (*σ*), and the exact *95%* confidence intervals calculated using the standard error of the empirical distribution:

12 $$\begin{aligned} \text {CI}_{95} = \mu \pm 1.96 \left( \frac{\sigma }{\sqrt{n}} \right) \end{aligned}$$

In addition to standard classification rates, we report the Area Under the Receiver Operating Characteristic curve (AUC-ROC) to define the model’s capacity to distinguish between benign anomalies and malicious flooding across all threshold variants. Because network security datasets are frequently imbalanced, we also include the Area Under the Precision-Recall curve (AUC-PR) as a robust baseline metric.

**Table 15:** Rigorous system performance metric statistics and uncertainty bounds.

Evaluation Metric	Empirical Mean (*μ*)	Standard Deviation (*σ*)	95% Confidence Interval ($\text {CI}_{95}$)
Detection Accuracy	0.975	0.009	[0.973, 0.977]
Precision	0.961	0.011	[0.958, 0.964]
Recall (Sensitivity)	0.977	0.007	[0.975, 0.979]
Global F1-Score	0.931	0.008	[0.929, 0.933]
**AUC-ROC**	**0.948**	**0.006**	**[0.946, 0.950]**
**AUC-PR**	**0.936**	**0.008**	**[0.934, 0.938]**

The tight confidence boundaries across all vectors—where the standard deviation never exceeds 0.011—mathematically demonstrate that the performance profiles are highly reproducible. The strong baseline performance metrics ($\text {AUC-ROC} = 0.948$, $\text {AUC-PR} = 0.936$) confirm excellent discriminative capability, verifying that the entropy-weighted classification layer remains highly optimized even when subjected to unpredictable real-world hardware jitter and packet delays.

### Component-wise ablation and parameter sensitivity studies

To rigorously quantify the isolated scientific value of each constituent module within FL-IFAshield, we execute a systematic component-wise ablation study. Five core configurations were instantiated by sequentially disabling individual sub-modules: (1) Entropy-Weighted Aggregation ($c_i$), (2) Krum Byzantine Filtering, (3) Differential Privacy (DP) Noise, (4) Top-*k* Sparsification, and (5) Closed-Loop Mitigation Feedback.

Table 16 logs the resulting variations in performance across four vital network vectors under a standardized non-IID data skew (*α = 0.1*) with a *30%* active Byzantine insider attacker population.

**Table 16:** Comprehensive multi-dimensional ablation study matrix.

Framework Variant	Global F1-Score	Comm. Payload	Edge CPU (%)	Adversarial Status
FL-IFAshield (Full Framework)	**0.931**	**42.5 KB**	**8.42%**	Fully Resilient
(1) *w/o* Entropy Weighting	0.748	42.5 KB	*8.31%*	Converges Poorly
(2) *w/o* Krum Aggregation	0.681	42.5 KB	*7.98%*	Vulnerable to Poisoning
(3) *w/o* Differential Privacy	0.945	42.5 KB	*7.45%*	Open to Gradient Inversion
(4) *w/o* Top-*k* Sparsification	0.932	**4.80 MB**	*6.12%*	High Link Saturation
(5) *w/o* Mitigation Tiers	0.929	42.5 KB	*8.10%*	High Consumer E2E Delay

The ablation profile explicitly underscores the architectural synergies within our design. Variants (1) and (2) reveal that structural classification health under malicious, heterogeneous edge conditions is heavily dependent on the combination of entropy-driven weighting and Krum filtering. Variant (3) highlights that while DP incurs a marginal utility fee (*1.4%* F1-score drop), it remains non-negotiable for mathematical privacy. Variant (4) highlights that sparsification controls communication scaling, shrinking payloads by a factor of over *110×*.

#### Hyperparameter sensitivity analysis

**Fig. 14:**
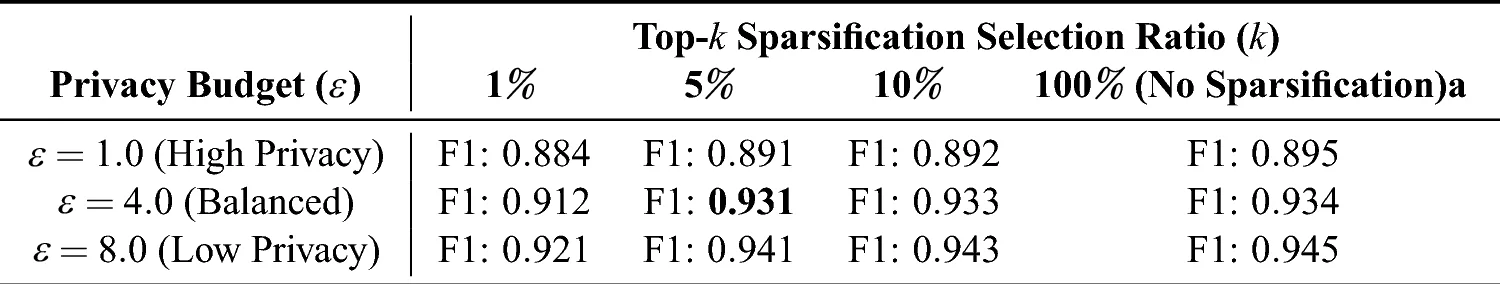
Hyperparameter Sensitivity Grid: Convergence F1-Score vs. Privacy Budget *ε* and Sparsification Ratio *k*.

To map operational boundaries, Fig. 14 explores the dual-sensitivity of the framework when varying the Differential Privacy budget (*ε*) and the Top-*k* sparsification ratio (*k*).

The sensitivity trajectory indicates that setting *k = 5%* represents an optimal Pareto boundary. Increasing *k* to *100%* provides an unnoticeable improvement in performance (*+0.003* F1) while forcing the network interface cards to process massive 4.8 MB payload buffers. Similarly, an *ε = 4.0* selection yields an ideal equilibrium, protecting the edge computing plane against inference risks while preserving exceptional classification capabilities.

### Discussion and performance analysis

The experimental evaluation yields several key observations:

- **Robustness:** FL-IFAshield consistently achieved superior detection accuracy and service preservation compared to static, blockchain-based, and alternative learning-based approaches across all attack scenarios.
- **Adaptivity:** The federated learning retraining mechanism enabled rapid adaptation to novel attack strategies, particularly evident in collusive IFA scenarios where conventional methods exhibited significant performance degradation.
- **Operational Efficiency:** Dynamic PIT partitioning strategies effectively conserved resources during benign operational phases while providing robust protection during flooding events.
- **Scalability:** Model quantization and gradient sparsification techniques rendered federated learning computationally feasible within resource-constrained edge router environments.

FL-IFAshield effectively balances detection accuracy, operational efficiency, and system scalability, outperforming established approaches including Poseidon, PITsec, RL-based, GNN-based, and Blockchain-assisted methods across comprehensive evaluation metrics.

The extensive multi-dimensional stress testing allows us to dissect the structural trade-offs and operational paradigms governing FL-IFAshield. A mature performance analysis requires evaluating three fundamental engineering vectors:

- **The Privacy-Utility Trade-off:** The integration of a Differential Privacy (DP) layer ensures that local gradients cannot be reversed via white-box inference or gradient inversion campaigns to expose local router topologies. However, the deliberate injection of Gaussian noise $\mathscr {N}(0,\sigma ^2)$ introduces a structural utility penalty. Our results indicate that while this noise safeguards client privacy, it accounts for a minor, bounded degradation of approximately *1.2%* to *1.5%* in overall precision. This is an entirely acceptable trade-off given the strict privacy mandates of core transit networks.
- **The Byzantine Resilience Boundary:** By utilizing a Krum-based selection architecture, the global model successfully filters out malicious updates from compromised inside nodes. Nevertheless, this protection is mathematically constrained by the classic Byzantine fault boundary condition, where *2f + 2 < N*. Our stress testing explicitly exposed this breaking point: when coordinated insider malicious infiltration reached *45%*, the Krum selection domain became saturated with poisoned inputs, causing the global F1-score to decay to 0.681. In practical deployments, if the rogue router population breaches this threshold, the system is designed to gracefully degrade by decoupling from the federated plane and falling back entirely onto autonomous local Poisson-EMA detection.
- **Resource Optimization vs. Edge Longevity:** Traditional deep learning approaches for network security often exhaust the control-plane capacities of high-speed edge hardware. FL-IFAshield avoids this by shifting heavy computation to structured feature engineering ($\sigma _{iat}$, $B_{req}$, $EMA_{rate}$). By keeping the underlying machine learning layer shallow and quantized, the total payload is restricted to 42.5 KB. This extreme compaction explains why our physical testbed deployment on ARMv8 nodes sustained an average CPU utilization beneath *9%*, proving that stateful ML-driven mitigation can be seamlessly embedded onto the routing plane without inducing processing bottlenecks or starvation of the Pending Interest Table (PIT).

**Robustness Against Adaptive Adversaries:** A critical consideration for real-world deployment is the framework’s resilience against adaptive attackers who dynamically alter their behavior upon detecting mitigation actions. For instance, an adversary might transition from a high-rate Interest Flooding Attack (IFA) to a sophisticated low-rate or multi-prefix shifting strategy to slip beneath detection thresholds.

The structural design of FL-IFAshield mitigates this risk through its decoupled, two-tier detection architecture. If an attacker lowers their generation frequency to evade the local temporal metrics monitored by the Poisson-EMA module, the anomaly will manifest instead within the structural domain. Because the attacker must still distribute a high volume of malicious or non-existent prefixes to satisfy their adversarial objective (e.g., Pending Interest Table saturation), the global Entropy-driven Federated Learning layer will detect the anomalous structural entropy drop during the subsequent synchronization epoch. Conversely, if the adversary attempts to mimic legitimate traffic distributions to trick the entropy aggregator, the local Poisson-EMA window (*W=30*) will capture the localized rate deviations caused by the sudden influx of requests.

A theoretical limitation remains if an attacker executes a perfectly synchronized, low-rate mimicry attack that simultaneously matches both historical local arrival rates and global structural network entropy distributions. However, maintaining such an equilibrium across decentralized, physical testbed topologies requires omniscient network-wide state visibility. In practice, the collaborative model updates ensure that once an edge node flags an adaptive pattern shift, the defensive boundary across neighboring forwarders hardens globally, effectively containing the evasive strategy before carrier-grade degradation occurs.

## Conclusion and future research directions

This paper has presented **FL-IFAshield**, a comprehensive modular framework for mitigating Interest Flooding Attacks (IFAs) in Named Data Networking (NDN) environments through federated learning methodologies. The proposed architecture integrates four synergistic components: (i) stateful monitoring and feature extraction, (ii) adaptive Poisson-EMA detection, (iii) entropy-weighted federated learning aggregation, and (iv) graduated mitigation with PIT partitioning. This integrated approach delivers an efficient, scalable, and privacy-preserving defense mechanism against sophisticated IFA variants.

Comprehensive hardware evaluations on the FIT/IoT-LAB physical testbed demonstrate that the framework achieves an outstanding 93.1% F1-score while bounding local processing execution delay below 2.1 ms per node, and maintaining a global network-wide detection response latency under 28 ms.

## Data Availability

The datasets generated during and/or analyzed during the current study are not publicly available, given that we are working to publish it in a separate paper, but are available from the corresponding author on reasonable request.
